# The N-Terminus of Murine Leukaemia Virus p12 Protein Is Required for Mature Core Stability

**DOI:** 10.1371/journal.ppat.1004474

**Published:** 2014-10-30

**Authors:** Darren J. Wight, Virginie C. Boucherit, Madushi Wanaguru, Efrat Elis, Elizabeth M. A. Hirst, Wilson Li, Marcelo Ehrlich, Eran Bacharach, Kate N. Bishop

**Affiliations:** 1 Division of Virology, MRC National Institute for Medical Research, The Ridgeway, Mill Hill, London, United Kingdom; 2 Department of Cell Research and Immunology, George S. Wise Faculty of Life Sciences, Tel Aviv University, Tel Aviv, Israel; 3 Division of Developmental Neurobiology, MRC National Institute for Medical Research, The Ridgeway, Mill Hill, London, United Kingdom; Fred Hutchinson Cancer Research Center, United States of America

## Abstract

The murine leukaemia virus (MLV) *gag* gene encodes a small protein called p12 that is essential for the early steps of viral replication. The N- and C-terminal regions of p12 are sequentially acting domains, both required for p12 function. Defects in the C-terminal domain can be overcome by introducing a chromatin binding motif into the protein. However, the function of the N-terminal domain remains unknown. Here, we undertook a detailed analysis of the effects of p12 mutation on incoming viral cores. We found that both reverse transcription complexes and isolated mature cores from N-terminal p12 mutants have altered capsid complexes compared to wild type virions. Electron microscopy revealed that mature N-terminal p12 mutant cores have different morphologies, although immature cores appear normal. Moreover, in immunofluorescent studies, both p12 and capsid proteins were lost rapidly from N-terminal p12 mutant viral cores after entry into target cells. Importantly, we determined that p12 binds directly to the MLV capsid lattice. However, we could not detect binding of an N-terminally altered p12 to capsid. Altogether, our data imply that p12 stabilises the mature MLV core, preventing premature loss of capsid, and that this is mediated by direct binding of p12 to the capsid shell. In this manner, p12 is also retained in the pre-integration complex where it facilitates tethering to mitotic chromosomes. These data also explain our previous observations that modifications to the N-terminus of p12 alter the ability of particles to abrogate restriction by TRIM5alpha and Fv1, factors that recognise viral capsid lattices.

## Author Summary

All retroviral genomes contain a *gag* gene that codes for the Gag polyprotein. Gag is cleaved upon viral maturation to release individual proteins, including matrix, capsid and nucleocapsid, providing the structural components of the virion. In murine leukaemia virus (MLV), Gag cleavage releases an additional protein, named p12, required for both early and late stages of the viral life cycle. The role of p12 during early events is poorly understood, and it is the only MLV protein without a function-associated name. Here, we show that p12 binds to the capsid shell of the viral core and stabilises it. Mutations that give rise to N-terminally altered p12 proteins result in a rapid loss of both p12 and capsid from viral cores, leading to abnormal core morphologies and abolishing the ability of particles to abrogate restriction by cellular factors that target viral capsid lattices. Understanding how the mature retroviral core forms and how it disassembles during infection is important as this determines the infectivity of all retroviruses, including HIV-1. Furthermore, altering core stability has recently become a novel target for HIV-1 therapeutics.

## Introduction

Retroviruses initially assemble as immature viruses containing a core of Gag and Gag-Pol polyproteins. During maturation these are cleaved into mature proteins by the virally encoded protease (PR). Cleavage of the gammaretrovirus Gag polyprotein produces four mature proteins: matrix (MA), p12, capsid (CA) and nucleocapisd (NC). A mass rearrangement follows cleavage, forming the mature CA core surrounding the condensed ribonucleoprotein complex [1]. Cryogenic electron microscopy studies on the maturation intermediates of HIV-1 have indicated that maturation is a step-wise and regulated process [2]. Maturation is essential for infectivity and blocking maturation using PR inhibitors has been heavily utilised in the control of HIV-1 infection [3]. Resistance to PR inhibitors remains a significant problem [4], and a greater understanding of the viral and cellular factors involved in maturation could yield new therapeutic targets. Furthermore, the CA shell of the core is beginning to be implicated in many early events from reverse transcription to integration [5]–[7], and understanding how the core is formed and maintained during an infection is of central importance.

**Figure 1:**
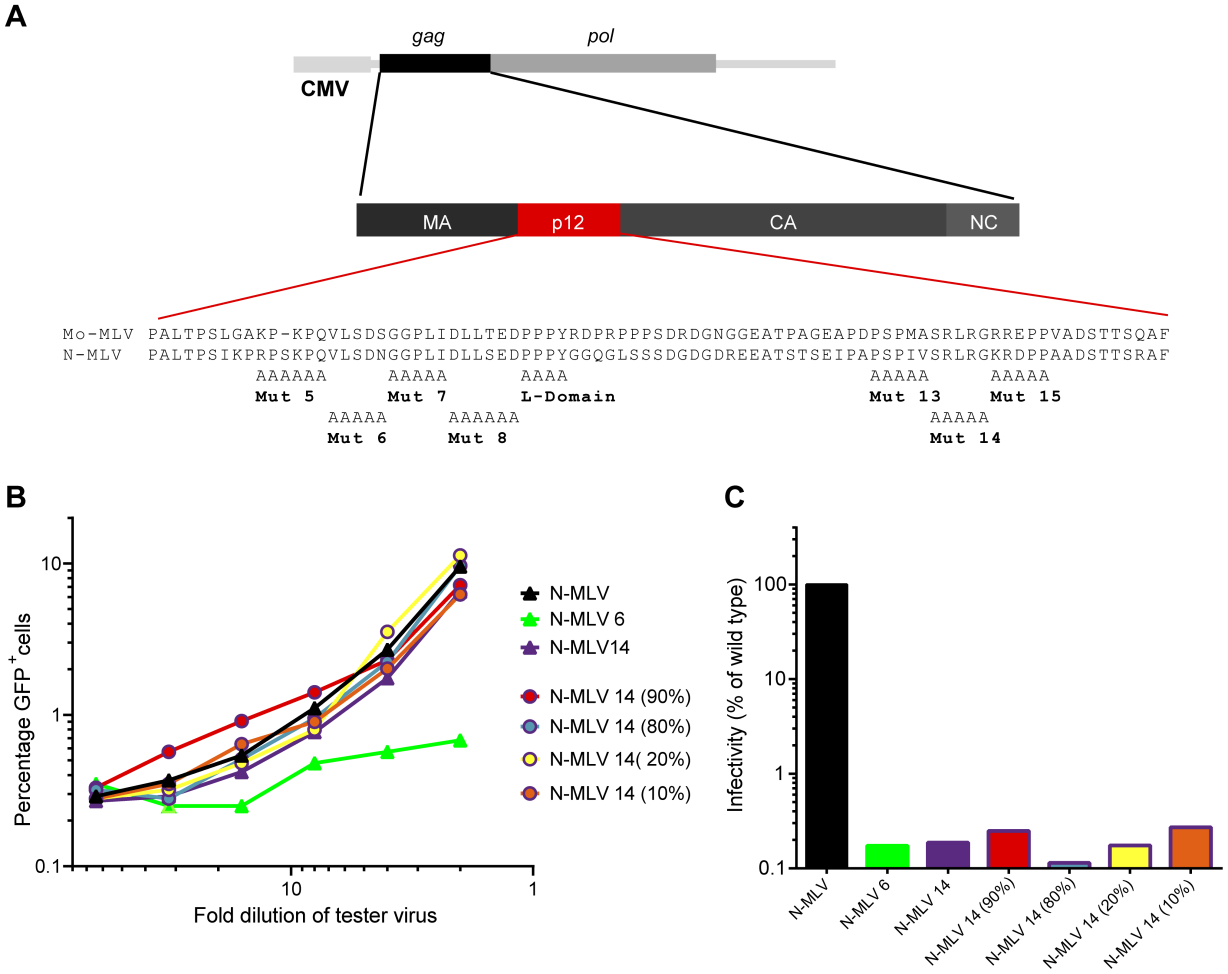
Abrogation of TRIM5alpha restriction by mixed p12 mutant particles. (A) Schematic representation of the Gag-Pol expression plasmid and p12 substitution mutants used in this study showing the amino acid sequence of Mo-MLV and N-MLV p12. (B) *LacZ*-encoding N-MLV VLPs containing either wild type p12, p12 mutant 6 or p12 mutant 14 (triangles), or a mixture of both mutants (circles) were synthesised. The percentage of p12 mutant 14 Gag-Pol expression plasmid in the transfection mix is indicated in brackets for the mixed mutants. Serial dilutions of these VLPs were used to challenge TE671 cells. Four hours later, cells were challenged with a fixed dose of GFP-encoding N-MLV VLPs, and after a further three days, the number of GFP positive cells was measured by flow cytometry. The percentage of GFP positive cells is plotted against *LacZ*-virus dilution. (C) D17 cells were challenged with equivalent RT-units of the VLPs used in (B). Infectivity was measured by detection of beta-galactosidase activity in a chemiluminescent reporter assay and plotted as a percentage of wild type N-MLV infectivity. These data are representative of multiple independent experiments.

The Gag protein p12 has important roles during both the early and late stages of murine leukaemia virus (MLV) infection [8]. It harbours the PPPY late-domain (L-domain), essential for recruiting HECT ubiquitin ligases to manipulate the ESCRT pathway for efficient budding [8], [9]. Additionally, seven mutants have been defined in Mo-MLV p12, four in the N-terminus and three in the C-terminus, which have a potent block during the early stages of infection (Fig. 1A) [8], [10]. The replication defects of these mutants fall into three groups: (i) mutants defective in reverse transcription *in vivo* (mutant 6), (ii) mutants defective in reverse transcription in certain cell lines (mutant 8) and (iii) mutants competent for reverse transcription but failing to integrate their viral DNA (mutant 5, 7, 13, 14 and 15) [8], [10]. We have recently shown that the N- and C- terminal regions of p12, mapped out by these mutants, are actually two sequentially acting domains, both of which are required to be active on the same p12 molecule for the transduction of target cells [10].

Biochemical analysis showed that isolated pre-integration complexes (PICs) from C-terminal p12 mutants were comparable to wild type, and were functional to integrate their DNA *in vitro* [11]. This suggested that the C-terminal domain mutants could be defective in accessing the host chromatin. We later corroborated this hypothesis by showing that p12 co-localised with mitotic chromatin during infection, a phenomenon not seen for the C-terminal p12 mutant 14 [12]. We and others have now shown that the infectivity defect of C-terminal p12 mutants can be rescued by the addition of heterologous chromatin binding sequences (CBS) into p12 [10], [13], [14]. Moreover, live imaging experiments with GFP-p12 labelled Mo-MLV have revealed that this rescue is mediated through restoration of the wild type chromatin tethering phenotype [14]. Gammaretroviruses favour integration into transcriptional start sites near CpG islands [15]–[17]. The co-localisation of p12 with chromatin suggested that it may in part determine integration site specificity. However, it was found that p12 chromatin tethering mediated by the addition of different CBS motifs did not alter the profile of integration site selection [13]. Thus, p12 function is more likely to retain the MLV PIC with the host chromatin, facilitating the interaction of integrase (IN) with BET-domain proteins [18]–[21].

Currently, little is known about the function of the N-terminal domain of p12. It has been shown that a ‘DLL’ motif in the N-terminus of p12 is involved in clathrin incorporation into the virion [22]. However, despite many retroviruses incorporating clathrin, the significance of clathrin incorporation remains unclear [22], [23]. Furthermore, most of our inactive N-terminal mutants are still able to bind clathrin (unpublished data), suggesting that failure to incorporate clathrin is not the reason they are inactive. We have recently shown that N-terminal p12 mutants were unable to saturate the CA-targeting restriction factors human TRIM5alpha and Fv1 in abrogation assays [10]. A number of possibilities exist as to why these p12 mutants have a defect in abrogation; therefore we set out to identify the cause of this phenotype.

Here, we show that mutations to the N-terminal domain of p12 alter the biophysical properties of the Mo-MLV CA core, an alteration evident before entry into the target cell. Analysis of the CA core morphology from N-terminal p12 mutants identified that p12 is required for mature CA core formation and stability of the core. Furthermore, the N-terminal domain of p12 is necessary for retaining p12 within the PIC and ensuring that it is present at the time of integration. Most importantly, we also show that p12 binds directly to MLV CA lattices, and suggest that this binding is required to stabilise the CA shell and prevent premature disassembly of the viral core.

## Results

### Only the N-terminal domain of p12 is required for abrogation of TRIM5alpha activity

One way of testing whether non-infectious viral particles can be recognised by the CA-targeting restriction factors Fv1 and TRIM5alpha is to perform a saturation, or abrogation, assay. Briefly, target cells expressing low levels of restriction factor are pre-exposed to increasing amounts of tester virus. If these tester virus particles can be recognised by the restriction factor, they will bind to and saturate the factor, abrogating restriction and allowing a GFP reporter virus, that would normally be restricted, to infect the cells. We previously demonstrated that p12 contains two domains that act in concert, one towards the N-terminus of the protein and the second towards the C-terminus [10]. Using restriction factor abrogation assays, we showed that N-tropic MLV (N-MLV) particles containing N-terminally mutated p12 lost the ability to abrogate Fv1 and TRIM5alpha whilst C-terminal p12 mutants did not (mutations shown in Fig. 1A) [10]. Interestingly, virus particles containing mixtures of both N- and C-terminally altered p12 were non-infectious showing that these mutations do not complement each other [10]. However, we wondered whether the inability of the N-terminal p12 mutants to abrogate restriction factors could be rescued in *trans* by C-terminal p12 mutant molecules. VSV-G pseudotyped *LacZ*-encoding virus-like particles (VLPs) were prepared by transfecting 293T cells with varying ratios of plasmids expressing either p12 mutant 6 or mutant 14 N-MLV Gag-Pol. Serial dilutions of these “tester” virus particles were added to TE671 cells, expressing humanTRIM5alpha, followed by a fixed and equal amount of GFP-encoding “reporter” N-MLV, and the number of GFP positive cells was measured after 72 hours. As expected [10], p12 mutant 6 VLPs were unable to abrogate restriction by TRIM5alpha, in contrast to the C-terminal mutant 14 and wild type particles (Fig. 1B, green triangles versus purple and black triangles, respectively). However, despite being non-infectious (Fig. 1C), all of the mixed particles tested, containing decreasing amounts of p12 mutant 14 from 90–10%, were able to abrogate TRIM5alpha restriction (Fig. 1B, coloured lines with open coloured circles). This phenotype was also observed when p12 mutant 6 was mixed with either of the other two C-terminal p12 mutants, 13 or 15 (Fig. S1 A and B). This suggests that, unlike for infectivity, C-terminal p12 mutant proteins can rescue the ability of particles containing N-terminal p12 mutant proteins to saturate TRIM5alpha, even when only 10% of the p12 molecules in the particle have a wild type N-terminus. Thus, a small amount of the N-terminus of wild type p12 is required to enable VLPs to interact with restriction factors.

### Mutations in the N-terminus of p12 alter the biophysical properties of the MLV CA core in target cells

**Figure 2:**
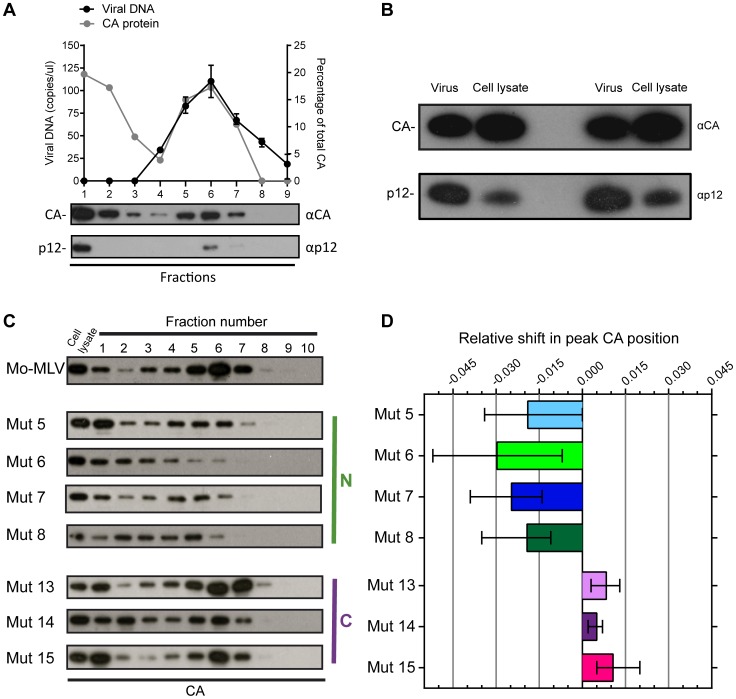
The effect of p12 mutations on the biophysical properties of the Mo-MLV RTC. (A) D17 cells were infected for four hours with wild type Mo-MLV, and cell lysates were separated using velocity sedimentation through a 10–42% (w/w) sucrose gradient. Fractions were analysed by qPCR with primers targeted to strong stop viral cDNA and proteins were precipitated and analysed by immunoblotting using anti-CA (top blot) and anti-p12 monoclonal (bottom blot) antibodies (Fraction 1 is the top of gradient). The graph displays the copies of viral DNA per microliter (black line) and the percentage of total CA in each fraction (grey line). (B) Wild type Mo-MLV was used to challenge D17 cells and after four hours the cells were lysed (cell lysate). An aliquot of the Mo-MLV VLPs used for the infection was also lysed (virus) and both samples were analysed by immunoblotting with anti-CA (top panel) and anti-p12 (bottom panel) monoclonal antibodies. Two repeats of this experiment are shown in the immunoblot. (C) Infected cell lysates from D17 cells challenged with wild type or p12 mutant Mo-MLV VLPs were separated using velocity sedimentation through a 10–42% (w/w) sucrose gradient and analysed by immunoblotting for CA. (D) For each experiment, the intensity of the CA signal on the immunoblot was determined and the relative shift in the peak CA position compared to wild type RTCs was calculated. The mean and range of at least three independent experiments are displayed.

TRIM5alpha targets the CA shell of N-MLV. The failure of N-terminal p12 mutants to abrogate restriction factors therefore suggests an intriguing possibility that the CA shell is perturbed in these mutants. Thus, we investigated what effect N-terminal p12 mutations had on CA. We previously showed that CA expressed from *gag* containing an upstream mutation in p12 could be incorporated into the CA core and be recognised by restriction factors, indicating that mutations in p12 do not affect the function of CA molecules *per se* [10]. Therefore, we studied the effect of p12 mutations on CA complexes using a modified fate-of-CA assay. D17 cells were challenged with wild type or p12 mutant Mo-MLV VLPs for 4 hours and cell lysates were separated on 10–42% (w/w) linear sucrose gradients by velocity sedimentation. The presence of CA in each gradient fraction was assessed by immunoblotting (Fig. 2).

For wild type particles, CA was present in two populations (Fig. 2A, top immunoblot). The first, in fraction 1 at the top of the gradient, presumably represented CA not in complexes, most likely released from viral particles after membrane fusion and/or during the poorly defined uncoating process. The second population was distributed through fractions 3 to 7 in the middle of the gradient, with the peak of material found in fraction 6. Importantly, this material co-migrated with viral cDNA, as measured by qPCR, (Fig. 2A, black line), and p12 (Fig. 2A, bottom immunoblot), implying that the CA complexes observed were reverse transcription complexes (RTCs). We have previously reported that in genetic assays, approximately only 10% of the p12 protein in the viral particle needs to be wild type for full infectivity [10]. CA and p12 are present at a 1∶1 ratio in viral particles as both are formed when Gag is cleaved. By directly comparing the ratio of CA to p12 in wild type viral particles and cell lysates four hours after infection (Fig. 2B), we could clearly see that much less p12 was present in the cell than CA. This suggests that most of the p12 is degraded rapidly after infection, presumably because it is not associated with the RTC, with no detrimental consequences for the virus.

When we analysed cell lysates following infection with our panel of p12 mutants, we observed that the pattern of CA migration through velocity sucrose gradients for the C-terminal p12 mutants was similar to wild type (Fig. 2C) with the peak in fractions 6 and 7. However, the N-terminal p12 mutants had a notably different CA distribution (Fig. 2C). Interestingly, overall, the CA complexes from the N-terminal mutants did not travel as far through the velocity gradient as the wild type complexes, indicating a reduction in their apparent S values. The phenotype of mutant 6 was the most strikingly different. Here, most of the CA was present in fractions 1 to 3 suggesting that the rate of sedimentation of these CA-containing complexes was much slower than wild type complexes. Notably, mutant 6 exhibits a 10-fold reduction in reverse transcription in cells [8], [10], and reverse transcription is known to be sensitive to the state and stability of the viral core. To quantify these observations and compare multiple experiments, the density of each fraction was measured and all gradients were found to be comparable. The intensity of the CA signal on the immunoblot was determined for each fraction by densitometry, and the fraction containing the most CA was identified as the “peak CA” fraction. The position of this peak CA fraction in the gradient for each p12 mutant was compared to that of wild type RTCs, and the relative shifts in peak positions are plotted in Fig. 2D. Clearly, the N-terminal p12 mutant RTCs had reduced sedimentation rates, suggesting that they have altered CA complexes. Interestingly, there was a slight, but reproducible, increase in the rate of sedimentation of the C-terminal p12 mutant CA complexes compared to wild type (Fig. 2D). Unfortunately, it was difficult to detect p12 in these samples due to the low levels of p12 in complexes and the fact that our monoclonal antibody to p12 does not recognise the N-terminal p12 mutants.

### N-terminal p12 mutants have intrinsically different CA complexes

**Figure 3:**
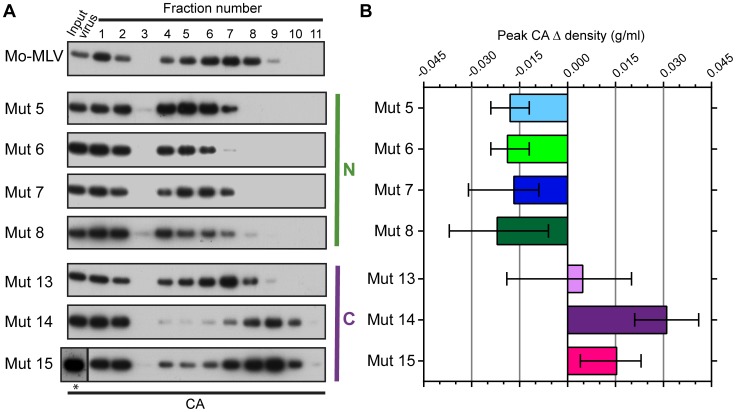
The effect of p12 mutations on the biophysical properties of the Mo-MLV intra-virion CA core. (A) Purified wild type or p12 mutant Mo-MLV VLPs were spun through a layer of detergent into a 10–42% (w/w) linear sucrose gradient. Fractions were harvested and analysed by immunoblotting using an anti-CA antibody (note: fractions 1–3 are diluted by 1∶13 to reduce the immunoblot signal, *: this input was run on a separate gel from the gradient fractions). Representative immunoblots are shown for wild type and each of the p12 mutants. (B) For each experiment, the sucrose density of the fraction containing the peak CA signal was measured, and the change in density compared to peak CA fraction for wild type virions was calculated. The mean and range from two independent experiments are displayed in the histogram.

Both the abrogation and biophysical data presented thus far have highlighted that N-terminal p12 mutant RTCs are altered in infected cells. However, to assess whether virions themselves are intrinsically different, we attempted to isolate CA cores from whole VLPs. Concentrated wild type and p12 mutant Mo-MLV VLPs were spun through a layer of detergent into a 10–42% (w/w) equilibrium sucrose gradient. Fractions were collected and the presence of CA analysed by immunoblotting. Firstly, it should be noted that the majority of CA was found in the first three fractions at the top of the gradient (samples were diluted 1∶13 relative to the remaining fractions before gel electrophoresis). The high level of un-complexed CA suggests that the detergent extraction was detrimental to core integrity, as has been described previously [24], although it is likely that a proportion of the CA in particles never forms part of the core. As was seen for the RTCs from infected cells (Fig. 2C), the N-terminal p12 mutant CA assemblies had a different distribution in the gradient to those from wild type Mo-MLV (Fig. 3A). For all samples, there was a population of CA at the top of the gradient (fractions 1–3) and a second population of CA in the middle of the gradient. However, the peak of CA was detected in fractions 6 to 8 for wild type virions, but in fractions 4 to 6 for all N-terminal mutants (Fig. 3A), indicating a reduction in density for these mutants. Curiously, the C-terminal p12 mutant CA assemblies migrated to a higher sucrose density than wild type assemblies (Fig. 3A). The density of the fraction containing the peak CA signal was measured for each mutant and the change in peak density compared to wild type particles was calculated. The mean and range from multiple independent experiments are plotted in Fig. 3B.

To ensure that the observed phenotype for the p12 mutant CA assemblies was not due to an altered biophysical property of whole virions, concentrated virus was analysed in identical sucrose gradients lacking detergent. The gradients were fractionated and the presence of CA detected by immunoblotting. The distribution of CA for all the p12 mutant virions was comparable to that of wild type Mo-MLV (Fig. S2A and B), suggesting that the composition of the mutant virions was unaltered. Therefore, taken together, these data suggest that mutation of the N-terminus of p12 alters the integrity or detergent susceptibility of the Mo-MLV CA core before entry to the target cell.

### The N-terminal domain of p12 is required to stabilise MLV CA core formation

**Figure 4:**
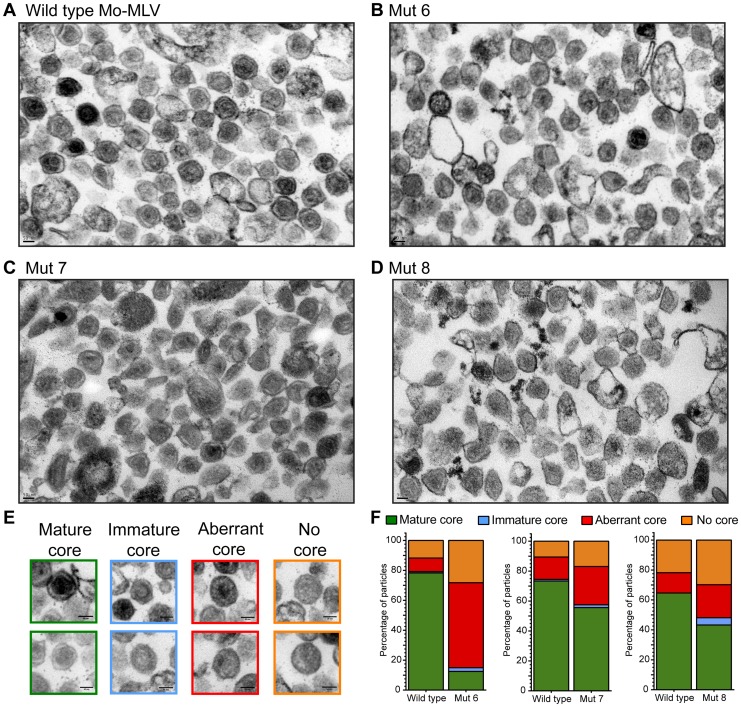
Analysis of the Mo-MLV p12 mutant intra-virion CA core by transmission electron microscopy. Purified Mo-MLV VLPs were pelleted and prepared for TEM. (A–D) Representative electron micrographs of wild type Mo-MLV and p12 mutant VLPs are shown. (E) Representative examples of the different core morphologies used to classify particles observed in the micrographs. (F) The morphology of all Mo-MLV particles (within 80–120 nm in diameter) were scored from multiple micrographs (at least 93 particles scored for each) and the results are displayed as a percentage of the total particles scored. All scale bars are 50 nm.

The altered distribution of the CA assemblies of N-terminal p12 mutants in sucrose gradients suggests that either the mutant viral cores are less stable or they are not correctly formed. To investigate core formation, we analysed the morphology of p12 mutant particles by transmission electron microscopy (TEM). Large batches of purified p12 mutant 6, 7, or 8 VLPs were pelleted and prepared for TEM alongside a matching wild type control. Thin (50 nm) sections were sliced through the virus pellet, stained and images acquired throughout the section at 20,000× magnification. Representative images are shown in Fig. 4 and further images are included in Fig. S3. The infectivity of the VLPs used was assessed in D17 cells (Fig. S4) and the three p12 mutants displayed the characteristic infectivity defect (100–1000-fold reduction in infectivity) as seen in Fig. 1C and previously described [10]. TEM images were selected at random, and the core morphologies of all VLPs with a diameter of 80–120 nm were classified into one of four categories according to a standard set of morphologies (Fig. 4E and F). At least 93 particles were scored for each sample. As expected, most wild type Mo-MLV virions contained a roughly circular electron-dense mature core filling up most of the intra-virion space (Fig. 4A and F, Fig. S3A). Strikingly, only 13% of p12 mutant 6 virions contained such cores compared to 78% of the corresponding wild type control (Fig. 4B and F, Fig. S3B). The majority of these particles either had no identifiable mature or immature core (28%) or contained cores with a grossly aberrant morphology (57%) such as a small electron dense spot or asymmetrical restricted density (Fig. 4B and F, Fig. S3B). The p12 mutant 6 particles manifest their defect earlier in the viral life cycle than the other p12 mutants; failing to synthesise wild type levels of cDNA in the target cell [8], [10]. In contrast, p12 mutant 7 is competent to reverse transcribe normally [8], [10]. Somewhat intuitively therefore, p12 mutant 7 had less of an effect on core morphology than p12 mutant 6, with 26% and 17% of virions containing an aberrant core or lacking a core, respectively (Fig. 4C and F, Fig. S3C). A much higher number of p12 mutant 7 virions contained a mature core (56%) when compared to p12 mutant 6, although this was still considerably lower than the corresponding wild type control (73%) (Fig. 4F). p12 mutant 8 has an interesting phenotype. It has been shown to have a defect in reverse transcription in NIH 3T3 cells [8], but was fully competent to synthesise viral cDNA in D17 cells [10]. Thus, it may be considered to have an intermediate phenotype between mutants 6 and 7 with regard to cDNA synthesis. When p12 mutant 8 virions were analysed by TEM, the range of core morphologies was similar to p12 mutant 7 (Fig. 4D and F, Fig. S3D). Only 22% of mutant 8 virions contained an aberrant core and 30% contained no identifiable core at all. Less p12 mutant 8 virions contained a mature core than p12 mutant 7 (43% vs 56%), although the corresponding wild type control also had a reduced number of virions with mature cores (64% vs 73%) (Fig. 4F). These observations suggest that mutation of the N-terminal domain of p12 is detrimental to the structure of the mature Mo-MLV CA core, and the severity of the defect correlates with the ability of particles to reverse transcribe in D17 cells.

### Alteration of the CA core is only evident after maturation

Gag and Gag-Pol are incorporated into the immature Mo-MLV particle as polyproteins, forming an immature Gag lattice. During maturation, the viral protease (PR) cleaves the polyproteins into individual Gag and Pol proteins, allowing CA to rearrange and assemble into a structurally distinct mature CA lattice [25]–[30]. Since p12 mutant 6 failed to assemble mature CA cores correctly, we asked whether p12 mutant 6 immature Gag lattices, the precursor of the mature core, were also altered.

**Figure 5:**
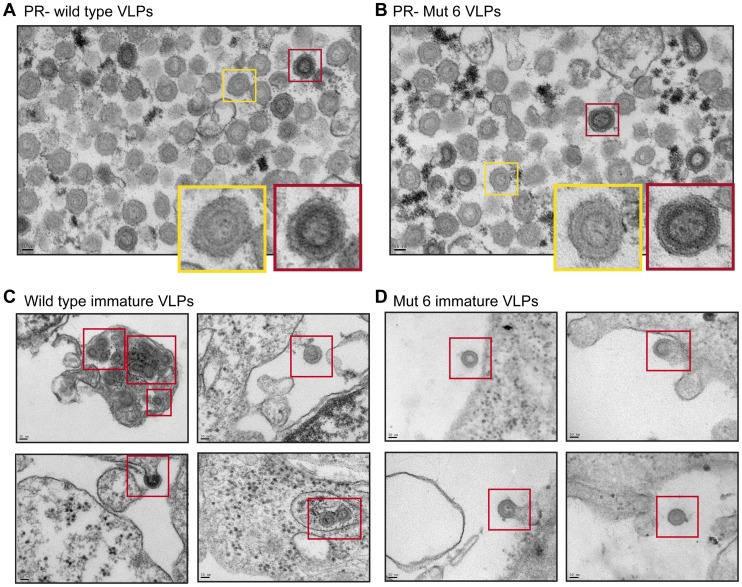
The effect of p12 mutant 6 on the immature Mo-MLV core. (A and B) A mutation was introduced into the viral protease (PR) to inactivate its activity (D32L, called PR-). PR- (A) wild type and (B) p12 mutant 6 Mo-MLV VLPs were prepared for TEM and representative electron micrographs of each are shown. The coloured boxes show enlargements of individual virions. (C and D) Wild type and p12 mutant 6 Mo-MLV producer cells were pelleted and prepared for TEM. Representative electron micrographs of (C) wild type and (D) p12 mutant 6 immature VLPs budding from producer cells are shown. Immature VLPs are highlighted by the red boxes. All scale bars are 50 nm.

Immature VLPs were produced by transfecting 293T cells with a Gag-Pol expressing construct that contained the D32L mutation in PR, which abolishes enzymatic activity [31]. Protease minus (PR-) wild type and p12 mutant 6 VLPs were concentrated and analysed by equilibrium sedimentation (Fig. S5). This analysis revealed no difference in the density of whole immature p12 mutants compared to the wild type control, as was seen for whole mature p12 mutant VLPs (Fig. S2), confirming that no gross abnormalities occur during particle assembly. Large batches of PR- wild type and p12 mutant 6 VLPs were then synthesised and prepared for TEM analysis as described above. Strikingly, PR- wild type and p12 mutant 6 VLPs were almost identical (Fig. 5A vs B). For both, nearly all particles contained an electron dense ‘train track’-like ring directly underneath the lipid bilayer, indicative of an immature core morphology [26]. Moreover, when cells producing either wild type or p12 mutant 6 VLPs with unmodified protease were analysed by TEM, “natural” immature particles could be seen budding from both p12 mutant 6 and wild type producer cells that were indistinguishable from one another (Fig. 5C vs D). Taken together, these data suggest that the p12 mutant 6 immature Gag lattice is not grossly altered; indicating that the core defect either manifests during maturation or is too subtle to be detected in immature particles.

### The N-terminal domain of p12 is required for retention of p12 in the MLV PIC

Although p12 mutant 6 has a clear defect in mature core formation, p12 mutants 7 and 8 appeared to produce significant amounts of particles (approximately half) with wild type-like core morphologies (Fig. 4). Therefore, the more than 99.5% reduction in infectivity of these mutants (Fig. S4) could not be explained by the complete absence of correctly formed mature cores. The data from our biophysical studies (Fig. 2 and 3) determined that the CA complexes are altered, so if this is not reflected by core appearance, it might suggest that the core is less stable. Moreover, mutations that alter the stability of the HIV core lead to aborted infections [32]. To observe cores *in vivo*, we followed infections by indirect immunofluorescence. Ecotropic Mo-MLV VLPs containing a previously described single myc-tag in p12 [12] were synthesised by transient transfection in 293T cells. U/R cells (U20S cells stably expressing mCAT-1 [12]) were challenged with equal doses of these wild type (MOI 3) or p12 mutant Mo-MLV-myc VLPs by spinoculation (1000×g) at 4°C. Cells were extensively washed, to remove unbound VLPs, returned to the incubator and fixed at various times post-infection. All the infections were done in duplicate with one sample stained for the myc-tagged p12 and the other for the CA protein as the fixing protocols necessary for each antibody were incompatible.

**Figure 6:**
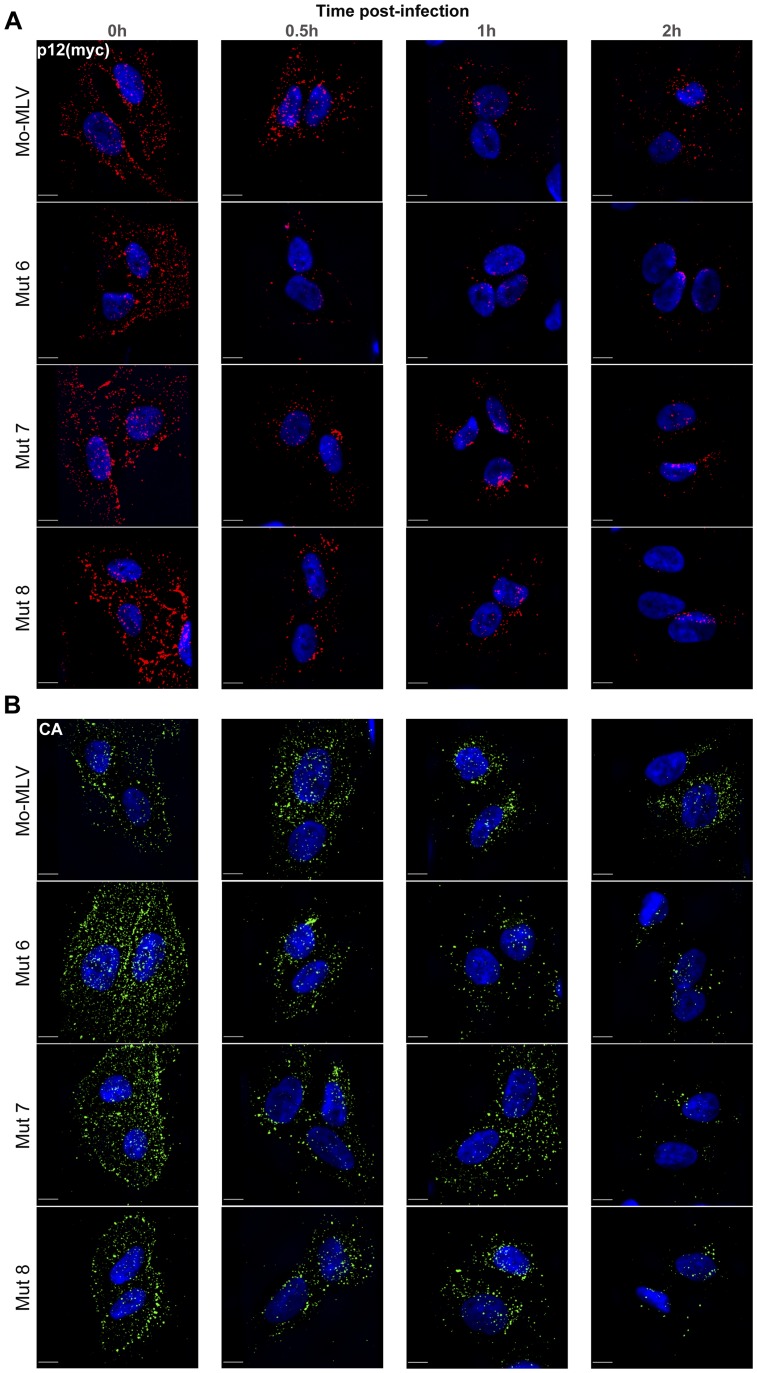
Immunofluorescence of p12 and CA in cells infected with Mo-MLV p12 mutants. U/R cells were challenged with ecotropic wild type or p12 mutant Mo-MLV VLPs, containing a myc-tag in p12, by cold spinoculation (MOI 3). Cells were fixed at various times post-infection and stained with either an (A) anti-myc or (B) anti-CA antibody followed by a Cy3 (A) or FITC (B) -conjugated secondary antibody. The nuclear DNA was counterstained using DAPI (blue). Images from the time course were captured using a spinning disk confocal microscope and representative images of cells from each time point are shown. All images are three dimensional acquisitions projected on a two dimensional plane. Images were processed using SlideBook. Scale bars are 10 µm.

For wild type Mo-MLV, an apparent decrease in the number of p12 puncta could be seen with time (Fig. 6A, top row). Traditionally, it has been thought that the rate of CA uncoating for MLV is slower than HIV-1, with significant amounts of CA remaining with the MLV PIC during transit to the nucleus [24], [33]. In line with these observations, we could see that cells challenged with wild type Mo-MLV contained large numbers of CA puncta, which remained for the length of the time course (Fig. 6B, top row). Thus, during infection with wild type Mo-MLV, some proportion of the viral p12 is shed from the PIC faster than CA, mirroring our earlier immunoblot observations (Fig. 2B). Interestingly, all of the N-terminal p12 mutants exhibited a differing phenotype to wild type Mo-MLV: A significant reduction in the number of p12 puncta could be observed very early, 0.5–1 hour post-infection (Fig. 6A and S6A). Furthermore, the number of CA puncta in N-terminal p12 mutant challenged cells also decreased with time (Fig. 6B and S6B). Consistent with our biophysical data, cells infected with the C-terminal p12 mutant 14 showed a similar pattern of puncta to wild type infections (Fig. S6A and B, bottom row).

**Figure 7:**
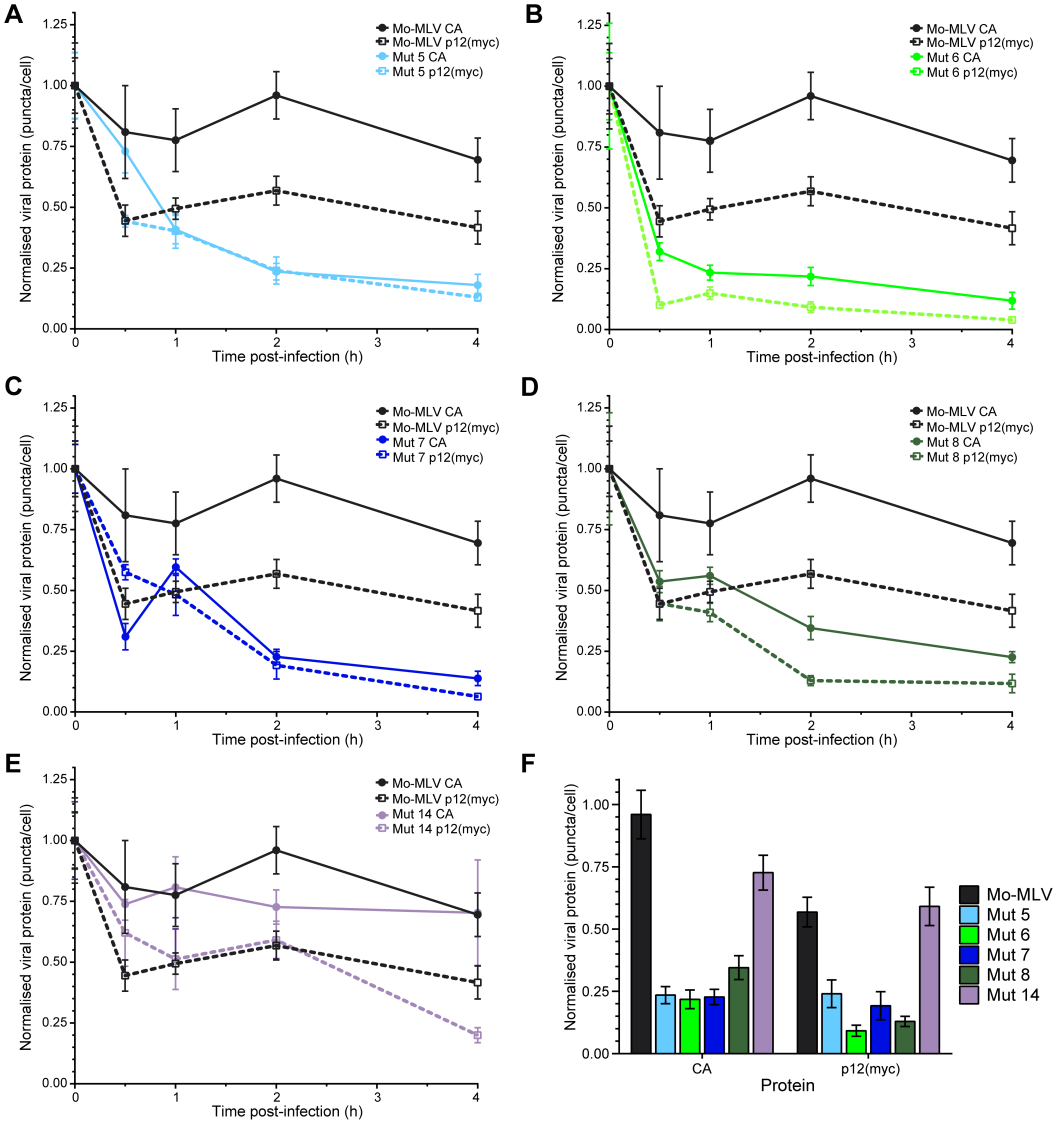
Kinetics of p12 and CA loss from cells infected with Mo-MLV. Ten cells were randomly chosen (based solely on nuclear stain) and imaged using a spinning disk confocal microscope. The numbers of p12(myc) or CA puncta in each cell were determined. The mean number of puncta per cell was normalised to the mean number of CA or p12(myc) puncta present at time point zero for each infection. The amount of p12 (dashed lines) and CA (solid lines) puncta are displayed against time in the graphs for: (A) p12 mutant 5, (B) p12 mutant 6, (C) p12 mutant 7, (D) p12 mutant 8 and (E) p12 mutant 14. Wild type Mo-MLV data (p12, black dashed and CA, black solid lines) are also plotted in each graph as a reference. Each point indicates the mean with SEM error bars. (F) The normalised numbers of CA and p12(myc) puncta in cells two hours post-infection are displayed as a bar chart showing the mean with SEM error bars.

To quantify these observations, cells were chosen at random using the nuclear counterstain and the entire cell body was imaged using a spinning disk confocal microscope. Outlines of the cells were drawn and the number of p12(myc) and CA puncta within each cell determined. Table S1 shows the mean numbers of puncta measured at time zero and two hours post-infection. The mean number of p12(myc) and CA puncta at each time point for each infection was normalised to the mean number of puncta at the zero hour time point and plotted against time post-infection. Figure 7 shows the analysis from 10 cells containing high numbers of puncta (∼250–300 puncta per cell at the zero hour time point). Importantly, very similar results were obtained from analysis of 10–16 cells from separate infections containing lower numbers of puncta; 30–60 puncta per cell at the zero hour time point (unpublished data). These analyses clearly demonstrate that there was a rapid reduction of p12 puncta from all N-terminal p12 mutant infected cells (Fig. 7A–D, coloured dashed lines), with more than 75% of the p12 puncta lost by two hours post-infection (Fig. 7F). In contrast, less than 50% of the p12 puncta were lost in cells infected with either wild type Mo-MLV (Fig. 7A–E, black dashed line, 7F) or p12 mutant 14 (Fig. 7E, purple dashed line, 7F). Notably, the number of CA puncta was also reduced by 65–75% by two hours post-infection in cells infected with the N-terminal p12 mutants (Fig. 7F), while there was only a minor reduction of CA puncta for wild type and p12 mutant 14 infections (Fig. 7F). Statistical analysis (t-test) of the number of puncta in cells two hours after infection (Fig. 7F) showed highly significant differences for N-terminal p12 mutants compared to wild type infections. Specifically, comparing p12 puncta with wild type gave p values of 0.01, 0.002, 0.0038 and 0.0029 for p12 mutants 5, 6, 7 and 8 respectively, and comparing CA puncta with wild type gave p values of 0.0028, 0.0023, 0.0029 and 0.0036 for p12 mutants 5, 6, 7 and 8, respectively. Taken together, these results suggest that alteration to the N-terminus of p12 results in a rapid loss of both p12 and CA itself from incoming viral cores. This suggests that the N-terminal domain of p12 is required for the retention of p12 within the RTC, and for conservation of the MLV CA core in the target cell.

### The N-terminus of p12 is responsible for binding to CA

**Figure 8:**
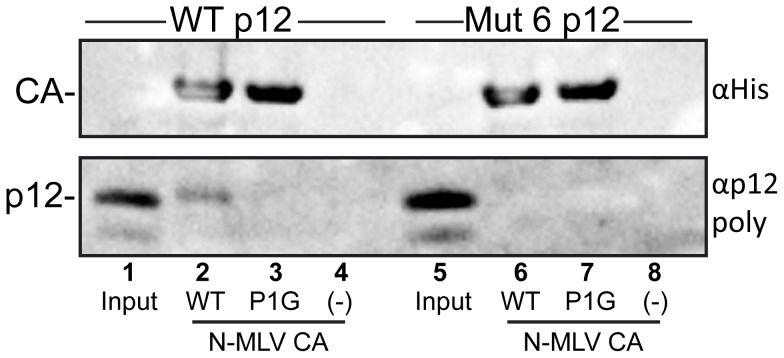
Binding of purified p12 to N-MLV CA-coated lipid nanotubes. Multimeric arrays of wild type (WT) and P1G mutant N-MLV CA were generated by immobilising the His-tagged purified proteins on lipid nanotubes comprising the Ni2^+^-chelating lipid, DGS-NTA. Purified wild type p12 (lanes 2–4) or p12 Mutant 6 (lanes 6–8) were incubated with lipid nanotubes coated with WT CA (lanes 2 and 6), P1G CA (lanes 3 and 7) or no tubes (lanes 4 and 8) prior to centrifugation through a sucrose cushion. The pelleted material was resuspended in SDS-PAGE sample buffer and probed for CA and p12 by immunoblotting, using appropriate antibodies. Input, lanes 1 and 5, represents 1/40^th^ dilution of the purified p12 proteins before incubation with CA-coated lipid nanotubes.

For p12 to be incorporated into the RTC, one would expect p12 to interact with core components. Indeed, a small amount of CA was previously immunoprecipitated from cells challenged with Mo-MLV using an antibody against myc-tagged p12, although this could not be recapitulated by immunoprecipitation of p12 from virions [12]. Given that p12 appears to influence the stability of the core, it is logical to predict that p12 binds directly to CA. However, a direct binding has never been shown, and most CA binding assays are hindered by the fact that the CA in the RTC is present in the form of a lattice, so monomeric CA may not recapitulate the binding surface present in an array. Fortunately, a protocol to form mature MLV CA lattice arrays on lipid nanotubes was previously established to study CA-Fv1 interactions [34]. We therefore used this approach to investigate whether p12 directly binds the CA lattice. Briefly, purified His-tagged N-MLV CA was immobilised on lipid nanotubes comprising the Ni2^+^-chelating lipid, DGS-NTA. These tubes were then incubated with purified p12 protein, and bound complexes were separated from unbound p12 by centrifugation through a sucrose cushion. The pelleted fraction was analysed for the presence of His-tagged CA or p12 proteins by immunoblotting with anti-His tag and anti-p12 polyclonal antibodies respectively. Fig. 8 shows representative immunoblots from 4 independent experiments that demonstrate detectable binding of wild type p12 protein to CA-coated lipid nanotubes (lane 2) but not p12 mutant 6 (lane 6). Importantly, we did not detect binding of either p12 protein to a version of CA that cannot form high density, regular arrays, CA-P1G [34] (lanes 3 and 7) showing there was little non-specific binding. Nor did either p12 protein pellet in the absence of CA-coated nanotubes under these conditions (lanes 4 and 8). In addition, cell lysates expressing either Fv1^b^ or Fv1^n^ were also incubated with the same CA-coated tubes as a positive and negative control for CA binding respectively [34]. Fig. S7 shows that we could detect binding of Fv1^b^ to the nanotubes, but Fv1^n^ had much reduced binding as expected (compare lanes 2 and 6), confirming that the CA was arranged in regular arrays that mimic true viral cores. Both Fv1 proteins showed weak binding to CA-P1G (lanes 3 and 7) indicating some non-specific binding. Together, this indicates that p12 does bind directly to the CA lattice and that the N-terminus of p12 is necessary for this interaction.

## Discussion

The p12 protein of MLV is essential during the early stages of viral replication [8], [10], [11], [35]. We previously showed that viruses bearing p12 proteins with alterations to their N-terminus were unable to abrogate restriction activity of TRIM5alpha or Fv1. There are various potential reasons for this (discussed in [10]), but as these restriction factors target the viral CA shell, this immediately suggested the intriguing possibility that mutations in p12 affected the viral core structure or stability. Influencing the properties of the core would explain the genetic interdependency between p12 and CA previously observed [10], [36]. Furthermore, as maintaining the appropriate core stability is vital for retroviral replication, through myriad plausible mechanisms (reviewed in [5]–[7]), this might also explain the infectivity defects of N-terminal p12 mutants. Therefore, we set out to investigate the interplay between the N-terminal domain of p12 and the MLV CA core.

First, we confirmed that only the N-terminus of p12 is important for the abrogation phenotype (Fig. 1B and S1). Previously, we discovered that mixing a small proportion (approximately 10%) of wild type p12 into a N-terminal p12 mutant particle was enough to restore infectivity and abrogation capability [10]. However, we were unable to rescue the infectivity of N-terminal p12 mutants using increasing ratios of C-terminally altered p12 (Fig. 1C and [10]). Despite this, these particles were able to saturate restriction factors (Fig. 1B and S1), confirming that neither infectivity nor the C-terminus of p12 is necessary for this effect.

The p12 protein is a functional constituent of the MLV RTC [12]. Accordingly, wild type p12 co-sedimented on sucrose gradients with CA and reverse transcribed cDNA from infected cells (Fig. 2A). In addition, there was a population of p12 that co-sedimented with un-complexed CA at the top of the gradient, suggesting that either a proportion of the p12 in the particle is not incorporated into the core, as is likely for CA, or that p12 is lost from the core after entry into the cell. CA and p12 are incorporated into the virus as part of the Gag polyprotein and are thus present in particles at a 1∶1 ratio. However, we consistently found it difficult to detect p12 by immunoblotting following velocity sedimentation of cell lysates. Consistent with our virological data that only a fraction of the p12 in the particles need be active [10], we observed a clear reduction in the amount of p12 present, compared to CA, in infected cell lysate (Fig. 2B). This suggests that RTCs contain less p12 than CA, and implies that any p12 not associated with the RTC is rapidly degraded. This presumed instability of free p12 is consistent with the failure to detect ectopically expressed p12 molecules in the absence of other viral proteins, unless tagged with a large, stable protein such as cherry fluorescent protein (unpublished data).

In a variety of different assays, we observed striking alterations in the characteristics of CA following mutation of p12. Analysis of N-terminal p12 mutant RTCs after separation of infected cell lysates on linear sucrose gradients revealed that they have a slower rate of sedimentation than wild type RTCs (Fig. 2C and D). The sedimentation coefficient of a particle, s, depends on its mass, density and shape, that impact on the frictional forces retarding the particle's movement. Thus, from our experiments, we can state that CA is present in different complexes in N-terminal p12 mutants and wild type viruses. However, this could reflect a difference in composition (a different collection of proteins), conformation (a different arrangement of proteins) or the relative amounts of individual proteins in the complexes. Notably, virions with reduced core stability would likely result in RTCs with slower sedimentation rates. Importantly, when we studied viral particles directly, the N-terminal p12 mutants had altered CA assemblies (Fig. 3), implying that this is not due to target cell factors or an effect on reverse transcription. Furthermore, analysis of p12 mutant viral particles by TEM also revealed differences in their CA core when compared to wild type particles (Fig. 4 and S3). It should be noted that wild type Mo-MLV particles displayed considerable heterogeneity in virus particle morphology (Fig. 4A and F, and S3A), as previously observed [37] and evident in the distribution of CA throughout the sucrose gradients (Fig. 2 and 3) [38]. This presumably reflects biological variation but is possibly enhanced by preparation of the samples for TEM. However, there was a considerable increase in particles with aberrant or absent cores for the p12 mutants, most strikingly for p12 mutant 6 (Fig. 4). Initial consideration of the TEM data suggested that p12 mutant 6 fails to form electron dense mature CA cores. However, approximately half the mutant 7 and 8 particles have formed mature cores, and so two alternative mechanisms for the function of p12 mutant 6 versus mutants 7 and 8 would need to be proposed. This does not seem credible, especially when all the N-terminal p12 mutants behave in a similar way in the biophysical assays (Fig. 2 and 3). Alternatively, mutations in p12 could result in a less stable core, with variation in the absolute stability depending on the N-terminal alteration. In the case of mutant 6, the core may be so unstable that it falls apart during formation or is more sensitive to disruption by preparation for TEM, while the other N-terminal mutants have a less severe core stability defect.

Further evidence for a core stability defect in p12 mutants comes from our immunofluorescence data. We could detect both p12 and CA containing complexes in cells challenged with wild type virus and N-terminal p12 mutants (Fig. 6 and S6). This implies that at least some fraction of CA and p12 are in complexes, even for p12 mutant 6 where the majority of particles contained minimal electron dense material (Fig. 4). In wild type particles, CA signal was slowly lost with time (unpublished data), likely due to uncoating and integration events. In keeping with our earlier observations, the p12 signal was lost faster than CA. However, we observed a more rapid loss of both p12 and CA puncta for the p12 mutants compared to wild type virus (Fig. 7). This suggests that the N-terminal domain of p12 is required for retention of p12 within the RTC, and indicates that the presence of p12 prevents premature loss of CA complexes. Thus, the core formed in the presence of N-terminal mutant p12 appears to be less stable.

As p12 has a role in nuclear retention and integration, and only a small proportion of the p12 in the virion is required for infectivity, it is possible that p12 is retained in the PIC by virtue of an interaction with integrase or the viral cDNA. Interestingly, allosteric integrase inhibitors have been shown to affect HIV-1 core morphology [39], suggesting that various viral components are involved in mature core formation. However, given the striking effects p12 mutation has on CA complexes, and the genetic evidence from chimeric viruses [10], [36], CA is the obvious binding partner for p12, and in particular the mature CA lattice. Using CA-coated lipid nanotubes, we were able to show that p12 does indeed bind to CA lattices but that there was no detectable binding for p12 mutant 6 (Fig. 8). A reduction in CA binding therefore correlates with the rapid loss of p12 puncta from cells and with the core morphology defects observed. Not only does this imply that p12 stabilises the CA lattice directly, but this is the first demonstration of direct protein binding to both MLV CA and p12.

As Gag proteins initially assemble into immature Gag lattices, we analysed the effect of p12 mutations on immature particles by equilibrium sedimentation and TEM. We observed no differences in the density (Fig. S5) or particle morphology between wild type and p12 mutant 6 virions, using either PR- mutants or imaging budding particles from producer cells (Fig. 5). Taken together with the fact that the mutants all produce similar numbers of particles to wild type MLV, and that individual Gag protein content is the same between wild type and p12 mutants by immunoblotting and ELISA [10], this implies that assembly and Gag processing are unaffected. However, there may be subtle alterations to these processes that we cannot detect, although the phenotype is certainly exaggerated post-maturation.

After proteolytic cleavage, the immature Gag lattice is thought to break down completely allowing the CA proteins to undergo a huge rearrangement [2], [25]. The current dogma is that CA alone determines the correct formation of the hexameric lattice structure seen in the mature core. Certainly, CA proteins from many retroviruses are able to form ordered hexameric lattices *in vitro*. However, in most systems, extra elements are present. For example, CA has often been fused to viral NC, and RNA has been introduced to seed the lattice formation [40], [41]. Arguably, CA fusions would represent immature lattices and not biologically mature core structures. Alternatively, CA arrays have been formed on lipid scaffolds using His-tagged CA proteins [34], [42], [43]. This has the advantage of increasing the avidity of CA interactions, perhaps relieving the need for additional catalysis or stabilisation. In addition, HIV-1 and RSV CA can be induced to form higher order assemblies by adding salt or crowding agents and/or altering pH [40], [44]–[47]. Nevertheless, many of these *in vitro* formed CA lattices have higher order structures, wide tubes or sheets, not observed in virions [41], [42], [45]. For HIV, a disulphide crosslinking strategy was deployed to enable purification and crystallization of soluble HIV-1 CA hexamers [48], [49], reinforcing the fact that stabilisation of the CA lattice requires some assistance. Perhaps the requirement for disassembly, or uncoating, of the CA shell necessitates the need for an inherently unstable mature CA lattice. However, it seems plausible that in virions, additional viral factors cooperate with CA to form a mature CA core of optimal stability. In the case of MLV, our data would suggest that p12 is required for this function. Moreover, p10 from Rous sarcoma virus (RSV), which is positionally analogous to p12 in Gag, has been shown to alter the morphology of CA-NC constructs formed *in vitro* from cylinders into spherical particles [41], [50]. Significantly, although viral production was somewhat reduced, alterations to the C-terminus of p10 also altered the mature RSV core morphology *in vivo* [51], [52].

Alpha-, beta-, gamma- and epsilon-retroviral genera all encode additional Gag cleavage products between MA and CA. Most of these are poorly characterised, but as mature CA lattices are thought to have similar arrangements in all retroviruses [42], it is tempting to speculate these additional Gag cleavage products function in an equivalent manner to MLV p12. Interestingly, one feature that they seem to have in common is that they harbour the late (L-) domain essential for viral budding [53], [54]. Although lentiviruses do not have an analogous protein between MA and CA, they too contain an L-domain. In the case of HIV-1, the L-domain is found in another protein, p6, cleaved from the end of Gag [53], [54]. L-domains therefore represent excellent examples of functional conservation despite little positional or sequence similarity. Moreover, despite different L-domains having alternative primary binding partners [53], [54], they have been shown to be functionally interchangeable [55]. Interestingly, HIV-1 p6 has been reported to alter core assembly by regulating CA processing [56]. Whether HIV-1 also contains a factor that stabilises the mature CA lattice remains to be seen; there is still a lot to learn about the formation and subsequent breakdown of the mature retroviral core structure. Nevertheless, the CA shell of the HIV-1 core has become an attractive drug target and alterations to core stability possibly influence immune responses to infection as well as local particle infectivity [57].

Overall, we have shown that p12 binds directly to CA lattices and that mutations in p12 that disturb this association have debilitating effects on the CA core, before the virus even infects a target cell. At its most striking, this defect manifests as failure to produce stable electron dense cores (mutant 6), and this correlates with a reduction in the ability of virions to reverse transcribe. Given the current notion that CA is vital for HIV integration events [5]–[7], it is tempting to speculate that an unstable core is responsible for the lack of integration seen for the N-terminal p12 mutants that can reverse transcribe normally (mutants 5, 7, and 8). Whilst this may be partly true, p12 is also required for chromatin tethering of the MLV PIC [10], [13], [14], and so we cannot discern whether the concomitant loss of p12 from the RTC or core instability itself is the cause of the infectivity defect and this will require further experimentation. Curiously, mutations to the C-terminus of p12 appeared to stabilise the CA complexes somewhat, particularly in virions (Fig. 2 and 3). Although the p12 proteins from C-terminal p12 mutants are still associated with the viral PIC and are present in the target cell nucleus, the viral cDNA is also unable to integrate. Determining the precise function of the C-terminus of p12 and identifying any C-terminal interaction factors may shed light on why CA assemblies from these mutants have altered phenotypes in our biophysical assays. Future work will endeavour to determine the interaction interface between p12 and the CA core to understand how the interaction affects core stability.

## Materials and Methods

### Plasmids and cloning

Three plasmids were co-transfected to synthesise retroviral VLPs: An envelope expression plasmid for either vesicular stomatitis virus G protein (pczVSV-G) [58] or the Mo-MLV ecotropic envelope (pMoSAF) [59]; a Mo-MLV-based retroviral vector encoding *LacZ* (pczLTR-LacZ) [60] or eGFP (pLNCG) [58], [61]; and either Mo-MLV (pKB4) [10] or N-tropic MLV (pCI G3N) [58] Gag-Pol expression plasmids. The generation of p12 mutations in these Gag-Pol expression plasmids has been described previously [10]. To create Mo-MLV Gag-Pol expression plasmids containing a previously described myc-tag in p12 [12], a *Bsr*GI-*Xho*I fragment from pNCS p12 1×MycR was swapped into pKB4 creating pKB4mycE. This was also done for p12 mutant 5 by cloning the same fragment from pNCS-PM5 p12 1×MycR into pKB4 creating pKB5mycE. To create the other p12 mutant Gag-Pol plasmids containing myc-tagged p12, site directed mutagenesis was performed on pKB4mycE using the Quik-Change kit (Stratagene). The following primers were used: p12 mutant 6 *for* 5′-*gccaaacctaaacctcaagctgctgctgccgctggggggccgctcatcga* and *rev* 5′- *tcgatgagcggccccccagcggcagcagcagcttgaggtttaggtttggc* ; p12 mutant 7 *for* 5′- *cctcaagttctttctgacagtgcggcggcggccgccgacctacttacagaagacccc* and *rev* 5′- *ggggtcttctgtaagtaggtcggcggccgccgccgcactgtcagaaagaacttgagg* ; p12 mutant 8 *for* 5′- *ggggggccgctcatcgccgcagctgcagcagcacccccgccttatagggacccaaga* and *rev* 5′- *tcttgggtccctataaggcgggggtgctgctgcagctgcggcgatgagcggcccccc* . To knock out protease (PR) activity in Mo-MLV VLPs, a single mutation was introduced into PR (D32L) using the Quik-Change kit with the following primers D32L *for* 5′-*gcaacccgtcaccttcctggtattaactggggcccaa* and *rev* 5′- *ttgggccccagttaataccaggaaggtgacgggttgc* . The resulting plasmids were called pKB4-PR^−^ for wild type p12, and pKB -5, -6, -7, -8, -13, -14 and -15 –PR^−^ for the p12 mutant Gag-Pol PR- expression plasmids respectively.

### Cells

293T, TE671, D17 and *M. dunni* cells (Bishop laboratory cell stocks) and U20S and U/R cells (Bacharach laboratory cell stocks) were maintained in DMEM (Invitrogen) supplemented with 10% heat inactivated foetal calf serum (Biosera) and 1% penicillin/streptomycin (Sigma), in a humidified incubator at 37°C and 5% CO2. U/R cells which stably express mCAT-1 [12] were maintained in the presence of 100 ug/ml Zeocin (Invitrogen).

### VLP production

Virus-like particles of Mo-MLV or N-MLV were prepared by co-transfection of 293T cells with a 1∶1∶1 ratio of three plasmids encoding the appropriate wild type or mutant Gag-Pol protein, VSV-G or MLV ecotropic Env, and a reporter gene (β-galactosidase or GFP) respectively, using polyethylenimine (PEI, PolySciences) as a transfection reagent. To make mixed mutant viral particles, two mutant N-MLV Gag-Pol expression plasmids were added to the transfection mix at different ratios, keeping the total concentration of Gag-Pol plasmid constant. After ∼24 hours, cells were washed and fresh media was added for a further ∼15 hours. Virus containing supernatants were harvested, filtered, and viral titres were quantified using a modified ELISA for reverse transcriptase activity (Cavidi). In the restriction factor saturation assays, Turbofect (Fermentas) was used as transfection reagent. Approximately 18 hours after transfection, cells were washed and sodium butyrate media (0.01 M sodium butyrate, 10% FCS and 1% penicillin/streptomycin in DMEM) was added for 6 hours before replacing with fresh media. VLPs were then harvested after ∼15 hours, as above. For qPCR experiments, viruses were treated with RQ1-DNase (Promega) prior to infection.

### Infections

VLP infectivity was determined as previously described [10]. Briefly, D17 cells were challenged with equivalent RT-units of *LacZ*-encoding VLPs. After 48–72 hours, the β- galactosidase activity in the cell lysate was measured using the Galacto-Star system (Applied Biosystems).

### Restriction factor saturation assays

Restriction factor saturation assays were performed as previously described [10]. Briefly, TE671 cells expressing endogenous human TRIM5alpha were infected with 2-fold serial dilutions of freshly harvested 293T cell supernatants containing *LacZ*-encoding VLPs. Cultures were incubated for 4–6 hours before adding a fixed amount of GFP encoding N-MLV. After 72 hours, infected cells were harvested and the percentage of GFP positive cells was determined by flow cytometry using a FACS Calibur analyzer (Becton Dickinson).

### Modified Mo-MLV fate-of-capsid assay

D17 cells were seeded at 1×10^6^ cells per well in a 6-well plate one day prior to infection. Each well was infected with 2 ml *LacZ*-encoding Mo-MLV VLPs (with 10 µg/ml polybrene) by spinoculation at 4°C (1600×g for 30 minutes). After 4 hours infection at 37°C, cells were washed and resuspended in 700 µl hypotonic buffer (10 mM Tris-HCl pH 8.0, 10 mM KCl, 1 mM EDTA supplemented with complete protease inhibitors). After 15 minutes incubation on ice, the cell suspension was applied to a Qiashredder column (Qiagen) and the subsequent cell lysate was layered on top of 10–42% (w/w) linear sucrose gradient (an aliquot of the cell lysate was kept for an input control). Samples were spun at 30,000 rpm for 45 minutes at 4°C in an SW55 rotor (Beckman Coulter). Fractions (500 µl) were collected from the top of the gradient using a syringe pump-driven gradient fractionator (Brandel). For each fraction, protein and viral DNA content were analysed using immunoblotting and quantitative PCR (qPCR) respectively. Proteins from each sucrose fraction were isolated by trichloroacetic acid precipitation and resuspended in 1× protein loading buffer.

### Biophysical analysis of virions in sucrose gradients

VLP containing cell supernatant was concentrated through a 20% (w/v) sucrose cushion in an SW41 rotor (Beckman Coulter) at 28,500 rpm for 90 minutes, 4°C. Supernatant and sucrose were aspirated and the viral pellet was resuspended in 250 µl PBS on ice for 4–5 hours. Linear 10–42% (w/w) sucrose gradients were formed in 14×89 mm polyallomer ultracentrifuge tubes (Beckman Coulter) with either: 250 µl of 1% Triton X-100 in 5% (w/w) sucrose PBS or just 5% (w/w) sucrose PBS (for intact VLP analysis) on top; followed by 250 µl of 2.5% (w/w) sucrose PBS. Concentrated virus was gently layered on top of the gradients (5 µl was kept for an input control), and spun in an SW41 rotor at 28,500 rpm for 16 hours at 4°C. Fractions were collected using a syringe pump-driven fractionator (Brandel) and each fraction was diluted in 4× protein loading buffer.

### Immunoblot analysis

All VLPs analysed by immunoblotting were concentrated by centrifugation through a 20% (w/v) sucrose cushion for 1 hour at 16,000×g, 4°C and resuspended in 1× protein loading buffer. Proteins were separated by SDS polyacrylamide gel electrophoresis (SDS-PAGE) and transferred onto polyvinylidene fluoride (PVDF, Millipore) membrane. Immunoblotting was performed with a rat anti-p30^CA^ (hybrodoma CRL-1912, ATCC), mouse anti-p12 monoclonal (hybrodoma CRL-1890, ATCC), goat anti-p12 polyclonal (a gift from J. Stoye), mouse anti-His (Penta·His Antibody, Qiagen) or rabbit anti-Fv1 (a gift from J. Stoye) followed by anti-rat, anti-goat, anti-mouse or anti-rabbit HRP-conjugated secondary antibodies. Detection was performed using the Immobilon chemiluminescent substrate (Millipore) and hyperfilm processed through a Fijifilm FPM-3800A developer.

### Quantitative PCR (qPCR) analysis

The quantity of viral DNA in each sucrose fraction was analysed using qPCR detection of minus strand strong stop reverse transcription products as described previously [10]. Briefly, reactions were performed in triplicate using Taqman Gene Expression Master Mix (Applied Biosystems) with 900 nM of each primer: oJWB45 (5′-*gcgccagtcctccgatagactga* ), oJWB47 (5′-*ctgacgggtagtcaatcactcag* ); and 250 nM of probe oJWB38 (5′-FAM- *atccgactcgtggtctcgctgttc* -TAMRA) [62]. The PCR reactions were performed with a Fast 7500 PCR system (Applied Biosystems) using standard cycling conditions: 50°C for 2 minutes, 95°C for 10 minutes followed by 40 cycles of 95°C for 15 seconds and 60°C for 1 minute. Relative cDNA copy number was determined by comparison to a dilution series of the LacZ-LTR plasmid in D17 cellular DNA.

### Transmission electron microscopy

Large batches of wild type and p12 mutant Mo-MLV VLPs were synthesised by transient transfection in 293T cells as described above with one modification: Transfected cells were washed and incubated in DMEM without added serum before viruses were harvested. VLP-containing 293T cell supernatant was pelleted by centrifugation at ∼100,000×g in an SW32 rotor (Beckman Coulter) using an Optima L-90K ultracentrifuge (Beckman Coulter). Pelleted VLPs were resuspended in 20 µm-filtered PBS on ice for 30 minutes. Resuspended viral pellets (300 µl total) were pooled and 300 µl of 5% gluteraldehyde/0.2 M sodium cacodylate buffer (Fisher Scientific and Sigma Aldrich, respectively) was added, mixed and fixed for 1 hour on ice. Virus was pelleted at 17,800×g for 1 hour at room temperature. The fixative was removed and 250 µl warm (37°C) 2% low melting point (LMP) agarose (Fisher Scientific) placed on top and mixed. Virus was then centrifuged at 16,200×g for 20 minutes in a centrifuge heated to 38°C, immediately followed by incubation on ice. After 30 minutes, ice-cold 2.5% gluteraldehyde/0.1 M sodium cacodylate buffer was layered on top of the solid agarose (up to the top of the tube). This was left overnight on ice to completely set the agarose. For VLP producer cell samples, approximately 2×10^7^ cells were washed from the culture dish with PBS and gently pelleted at 500×g for 5 minutes in a bench top centrifuge (4°C). The cell pellet was gently resuspended in 2.5% gluteraldehyde, 0.1M sodium cacodylate and pelleted at 600×g for 10 minutes (4°C). Fixation was continued overnight on ice.

Both the cell pellet and the VLP pellet set in LMP agarose were post-fixed with 1% osmium tetroxide for 90 minutes and washed with 0.1M sodium cacodylate. Samples were then stained with 1% aqueous uranyl acetate for 90 minutes and dehydrated in an ethanol series before propylene oxide. All samples were then embedded in medium Agar 100 resin and polymerised overnight at 70°C. 50 nm thick sections were stained with saturated ethanolic uranyl acetate and Reynold's lead citrate. Samples were viewed on a Jeol 1200EX transmission electron microscope (Jeol Ltd) operating at 80 kV and a magnification of ×20,000. Images of cells and purified particles were captured on an Orius CCD camera (Gatan) using the auto exposure mode. Quantification of core morphology from purified VLPs was performed on randomly selected micrographs and only particles between 80–120 nm in diameter were scored (at least 93 individual particles were scored for each sample).

### Indirect immunofluorescence

U/R cells were seeded at 4×10^4^ on sterile 13 mm coverslips (VWR) in a standard 12-well plate (Corning). Cells were challenged 16 hours later with ecotropic wild type or p12 mutant Mo-MLV VLPs at an MOI 3 (mutants were normalised to wild type by the level of RT activity) by spinoculation (1000×g at 4°C for 2 hours). Infections were done in duplicate from a single batch of diluted virus due to the differing antigen retrieval conditions required for the p12(myc) and CA antigens. Infected cells were then washed three times with pre-warmed DMEM complete and fixed at the indicated time points as follows: (i) for p12(myc) detection; 4% paraformaldehyde (AlfaAesar) for 20 minutes then 0.1% triton X100 in PBS for 10 minutes or (ii) for CA detection; 4% paraformaldehyde 2 minutes followed by −20°C methanol for 5 minutes. Cells were washed three times with PBS and blocked in 5% bovine serum albumin (BSA, Fisher Scientific) for 1 h. Primary monoclonal antibodies (hybridoma supernatants) were diluted 1∶6 in 1% BSA: mouse anti-myc 9E10 [12] or rat anti-p30^CA^ (CRL-1912, ATCC); and incubated on the cells for 1 hour. Coverslips were then washed with PBS three times for 10 minutes each and secondary antibodies diluted in 1% BSA were added for 1 hour. The goat anti-mouse Cy3-conjugated or goat anti-rat FITC-conjugated antibodies (Jackson Immunoresearch Laboratories) were diluted 1∶500 or 1∶100, respectively. DAPI stain was added to the slides together with the secondary antibodies.

### Fluorescence microscopy

Images were acquired with either a spinning disk confocal (Yokogawa CSU-22 Confocal Head) microscope (Axiovert 200 M, Carl Zeiss MicroImaging) or an Ultraview spinning disk confocal microscope (Perkin Elmer) equipped with a C9100-13 electron multiplying charged-coupled device (EMCCD, Hamamatsu). For quantification, the entire cell volume was imaged by confocal microscopy and the picture was deconvolved using the Nearest Neighbors deconvolution algorithm of SlideBook. Subsequently, three dimensional acquisitions were projected on a two dimensional plane. After this, the specific signals of the p12-based and CA-based staining were identified through intensity based segmentation and the number of objects (puncta) in the inspected cells was determined. Approximately 250 dots of p12 or CA signal per cell at time zero were analysed. All the above steps were performed using the SlideBook software (Intelligent Imaging Innovations).

### Recombinant protein expression and purification

Wild type and mutant 6 p12 sequences from N-tropic MLV were cloned into pGEX6.1 using the *Bam*HI and *Xho*I restriction endonuclease sites. The N-terminally GST-tagged fusion proteins were expressed in *E. coli* Rosetta 2(DE3)pLysS by inducing a mid-log culture grown in the presence of 1% glucose with 1 mM isopropyl-β-D-thiogalactopyranoside (IPTG). For lysis, cells were resuspended in 50 mM Tris pH 8, 500 mM NaCl, 0.5 mM TCEP, 0.1% Triton X-100 (Buffer A) in the presence of protease inhibitors (Roche) and incubated with Lysozyme (Sigma Aldrich) and Benzonase nuclease (Sigma Aldrich) for 1 hour at 4°C. The crude lysates were then sonicated twice for 5 minutes at 40% amplitude and centrifuged at 48,000×g for 45 minutes to remove debris. The clarified lysates were applied to 1 ml GST-trap columns (*GE Healthcare*). After washing with Buffer A, untagged-p12 was eluted from the resin by digestion with 3C precision protease. The eluate was then heated at 65°C for 10 minutes and centrifuged at 40,000×g for 20 minutes to remove precipitates. Acetic acid (pH ∼3) was added to the supernatant which was then centrifuged at 40,000×g for 20 minutes to remove DNA. The supernatant was then applied to a Superdex 75 (16/60) size exclusion column equilibrated in 200 mM Ammonium bicarbonate. Eluate fractions containing p12 were pooled and lyophilised. The purity of the protein preparations were assessed by SDS-PAGE and the concentrations were determined from the absorbance at 280 nm.

C-terminally His-tagged N-MLV CA WT and P1G mutant proteins were expressed and purified as previously described [34].

### Binding assays with CA-coated lipid nanotubes

The assays were performed essentially as previously described [34]. Lipid nanotubes were generated by combining the tube-forming lipid, d-galactosyl-β-1,1′ *N*-nervonoyl-d-erythro-sphingosine (GalCer) (*Avanti*) with the Ni^2+^-chelating lipid, DGS-NTA (*Avanti*) in a 7∶3 ratio. After mixing the lipids, residual chloroform and methanol were removed under a gentle stream of nitrogen and the lipids were resuspended by sonication in 10 mM Tris-HCl pH 8, 10 mM KCl, 100 mM NaCl, to a concentration of 0.5 mg/ml. The tubes were coated by incubating with 2 mg/ml of purified His-tagged N-MLV wild type CA or P1G CA mutant at a ratio of 1∶3 with 10 mM imidazole, for 1 hour at room temperature. Purified N-MLV p12 wild type and mutant 6 proteins were diluted to approximately 5 µg/ml, in dilution buffer (10 mM Tris-HCl pH 8, 10 mM KCl, 100 mM NaCl, 10 mM imidazole, 1% BSA). *M. dunni* cells expressing either Fv1^b^ or Fv1^n^ were lysed and cell lysates were diluted to 0.1 mg/ml total protein in dilution buffer. In each binding reaction, 200 µl of p12 or Fv1-containing cell lysate was incubated with 4 µl of CA-coated lipid nanotubes, for 2 hours at room temperature with gentle agitation. The samples were then layered on top of a 2 ml cushion of 40% (w/v) sucrose in 10 mM Tris-HCl pH 8, 10 mM KCl, 100 mM NaCl and centrifuged at 34,000×g for 1 hour at 4°C. The supernatants were then aspirated and the pellets were resuspended in 40 µl of 1× protein loading buffer. His-tagged CA, p12 or Fv1 in the pellet fractions were detected by immunoblotting.

## Supporting Information

Figure S1 **Abrogation of TRIM5alpha restriction by mixed p12 mutant particles.** *LacZ*-encoding N-MLV VLPs containing either (A) p12 mutant 6, p12 mutant 13 (triangles), or a mixture of both mutants (circles) or (B) p12 mutant 6, p12 mutant 15 (triangles), or a mixture of both mutants (circles) were synthesised. The percentage of p12 mutant 13 or 15 (For (A) and (B) respectively) Gag-Pol expression plasmid in the transfection mix is indicated in brackets for the mixed mutants. Serial dilutions of these VLPs were used to challenge TE671 cells. Four hours later, cells were challenged with a fixed dose of GFP-encoding N-MLV VLPs, and after a further three days, the number of GFP positive cells was measured by flow cytometry. The percentage of GFP positive cells is plotted against *LacZ*-virus dilution. These data are representative of multiple independent experiments.
(TIF)

Figure S2 **Migration profile of intact mature p12 mutant VLPs in an equilibrium gradient.** (A) Purified VLPs were subjected to equilibrium sedimentation through a 10–42% (w/w) sucrose gradient (without detergent). Fractions were collected and analysed by immunoblotting using an anti-CA antibody. Representative immunoblots are shown (Fraction 1 is the top of the gradient). (B) For each experiment, the sucrose density of the fraction containing the peak CA signal was measured, and the change in density compared to peak CA fraction for wild type virions was calculated. The mean and range of two independent experiments are displayed in the histogram.
(TIF)

Figure S3 **Additional electron micrographs of purified wild type and p12 mutant VLPs.** Mo-MLV VLPs were purified and prepared for TEM, as in Fig. 4. Additional electron micrographs of (A) wild type, (B) p12 mutant 6, (C) p12 mutant 7 and (D) p12 mutant 8 VLPs are shown. All scale bars are 50 nm.
(TIF)

Figure S4 **The infectivity of p12 mutant VLPs used for transmission electron microscopy.** D17 cells were challenged with equivalent RT-units of the wild type and p12 mutant *LacZ*-encoding VLPs used in the TEM analysis (Fig. 4 and S3). Infectivity was measured by detection of beta-galactosidase activity in a chemiluminescent reporter assay and plotted as a percentage of wild type N-MLV infectivity. (A) Wild type and p12 mutant 6 VLPs, (B) Wild type and p12 mutant 7 VLPs and (C) Wild type and p12 mutant 8 VLPs.
(TIF)

Figure S5 **Migration profile of intact immature p12 mutant VLPs in an equilibrium gradient** A mutation was introduced into PR to inactivate its activity (D32L, called PR-). (A) PR- wild type and p12 mutant VLPs were subjected to equilibrium sedimentation through a 10–42% (w/w) sucrose gradient (without detergent). Fractions were collected and analysed by immunoblotting using an anti-CA antibody. Representative immunoblots are shown (Fraction 1 is the top of the gradient). (B) For each experiment, the sucrose density of the fraction containing the peak CA signal was measured, and the change in density compared to peak CA fraction for wild type virions was calculated. The mean and range of three independent experiments are displayed in the histogram.
(TIF)

Figure S6 **Immunofluorescence of p12 and CA in cells infected with Mo-MLV p12 mutant 5 and 14.** U/R cells were challenged with ecotropic wild type, p12 mutant 5 or p12 mutant 14 Mo-MLV VLPs, containing a myc-tag in p12, by cold spinoculation (MOI 3). Cells were fixed at various times post-infection and stained with either an (A) anti-myc or (B) anti-CA antibody followed by a Cy3 (A) or FITC (B) -conjugated secondary antibody. The nuclear DNA was counterstained using DAPI (blue). (C) Images of uninfected U/R control cells fixed and stained as in (A) and (B). Images from the time course were captured using a spinning disk confocal microscope and representative images of cells from each time point are shown. All images are three dimensional acquisitions projected on a two dimensional plane. Images were processed using SlideBook. Scale bars are 10 µm.
(TIF)

Figure S7 **Binding of Fv1 to N-MLV CA-coated lipid nanotubes.** Multimeric arrays of wild type (WT) and P1G mutant N-MLV CA were generated by immobilising the His-tagged purified proteins on lipid nanotubes comprising the Ni2^+^-chelating lipid, DGS-NTA. Cell lysates containing Fv1^n^ (lanes 2–4) or Fv1^b^ (lanes 6–8) were incubated with lipid nanotubes coated with WT CA (lanes 2 and 6), P1G CA (lanes 3 and 7) or no tubes (lanes 4 and 8) prior to centrifugation through a sucrose cushion. The pelleted material was resuspended in SDS-PAGE sample buffer and probed for CA and Fv1 by immunoblotting, using appropriate antibodies. Input, lanes 1 and 5, represents 1/16^th^ dilution of cell extracts before incubation with CA-coated lipid nanotubes.
(TIFF)

Table S1 **Numbers of CA and p12 puncta counted in immunofluorescent studies.** The mean number of CA or p12 puncta present per cell at time zero or 2 hours post-infection is shown +/− standard errors. The numbers of puncta at 2 hours post-infection were normalised to the numbers of puncta at time zero and these data are represented graphically in the bar chart in Figure 7F.
(DOCX)
