# Supplementary figures and images for: The N-Terminus of Murine Leukaemia Virus p12 Protein Is Required for Mature Core Stability
Source: PLoS Pathog. 2014 Oct 30;10(10):e1004474. doi: 10.1371/journal.ppat.1004474 (PMC4214797; doi:10.1371/journal.ppat.1004474)

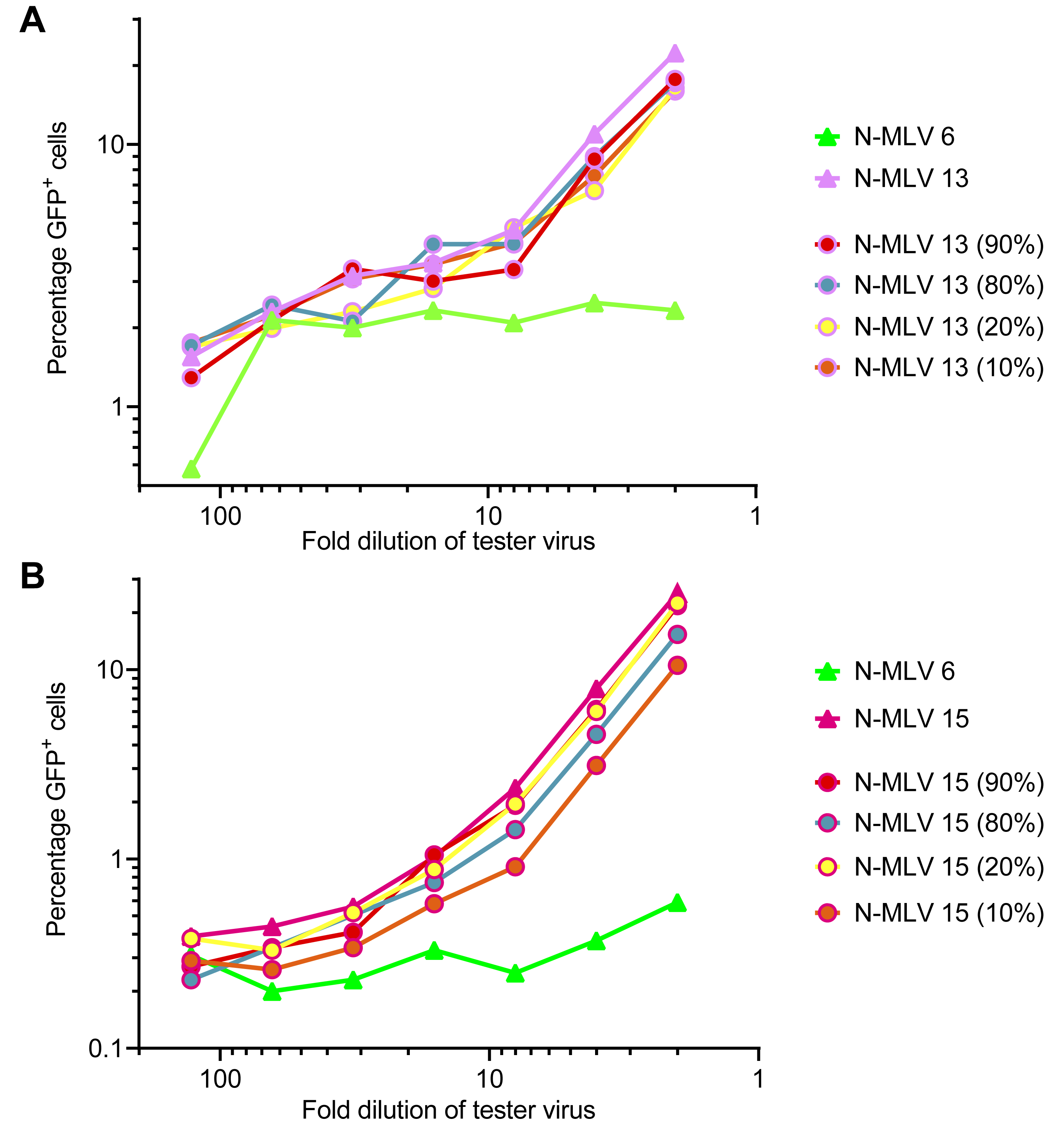

Supplement: Figure S1 — Abrogation of TRIM5alpha restriction by mixed p12 mutant particles. LacZ-encoding N-MLV VLPs containing either (A) p12 mutant 6, p12 mutant 13 (triangles), or a mixture of both mutants (circles) or (B) p12 mutant 6, p12 mutant 15 (triangles), or a mixture of both mutants (circles) were synthesised. The percentage of p12 mutant 13 or 15 (For (A) and (B) respectively) Gag-Pol expression plasmid in the transfection mix is indicated in brackets for the mixed mutants. Serial dilutions of these VLPs were used to challenge TE671 cells. Four hours later, cells were challenged with a fixed dose of GFP-encoding N-MLV VLPs, and after a further three days, the number of GFP positive cells was measured by flow cytometry. The percentage of GFP positive cells is plotted against LacZ-virus dilution. These data are representative of multiple independent experiments. (TIF) [file ppat.1004474.s001.tif]

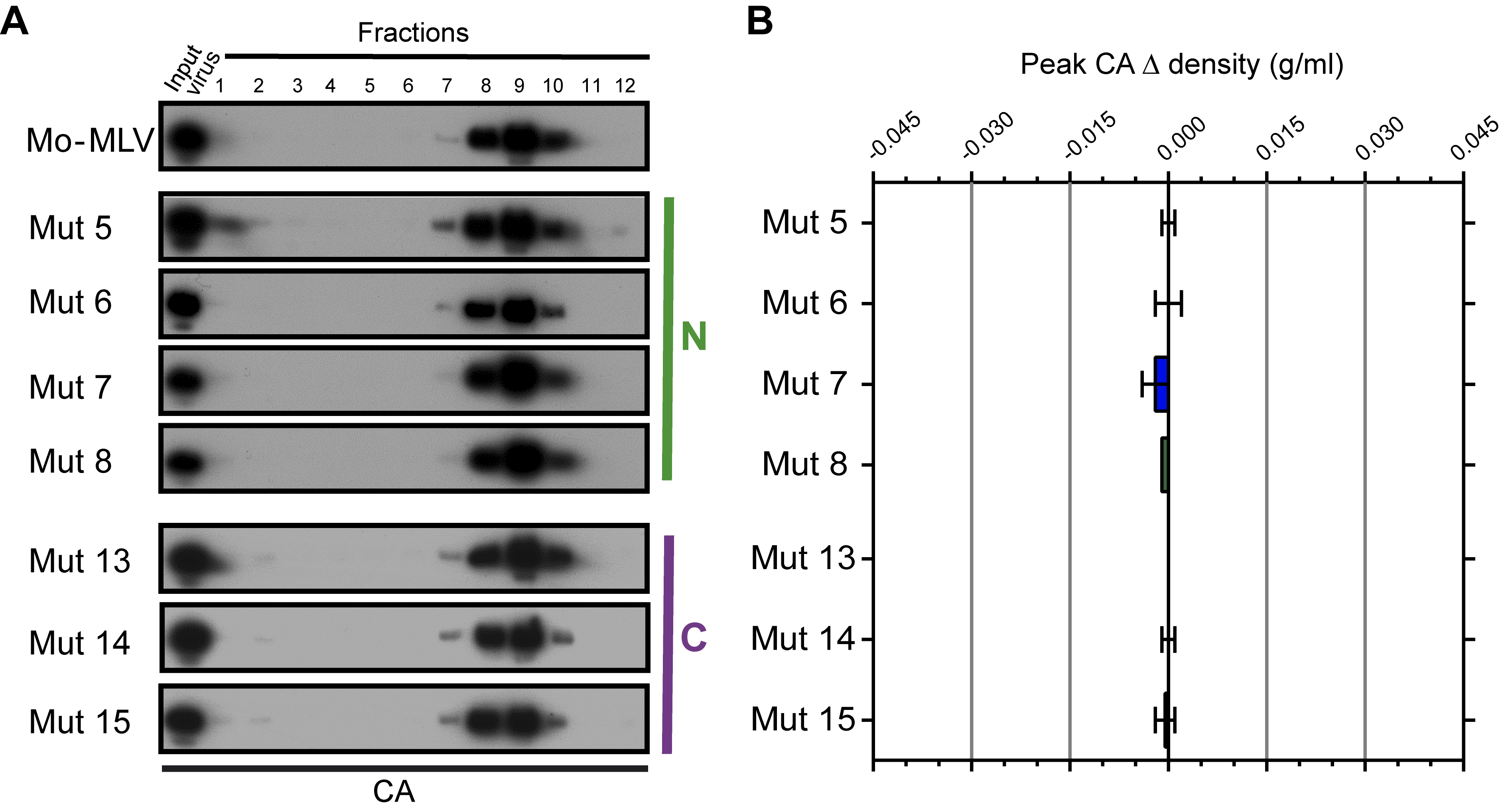

Supplement: Figure S2 — Migration profile of intact mature p12 mutant VLPs in an equilibrium gradient. (A) Purified VLPs were subjected to equilibrium sedimentation through a 10–42% (w/w) sucrose gradient (without detergent). Fractions were collected and analysed by immunoblotting using an anti-CA antibody. Representative immunoblots are shown (Fraction 1 is the top of the gradient). (B) For each experiment, the sucrose density of the fraction containing the peak CA signal was measured, and the change in density compared to peak CA fraction for wild type virions was calculated. The mean and range of two independent experiments are displayed in the histogram. (TIF) [file ppat.1004474.s002.tif]

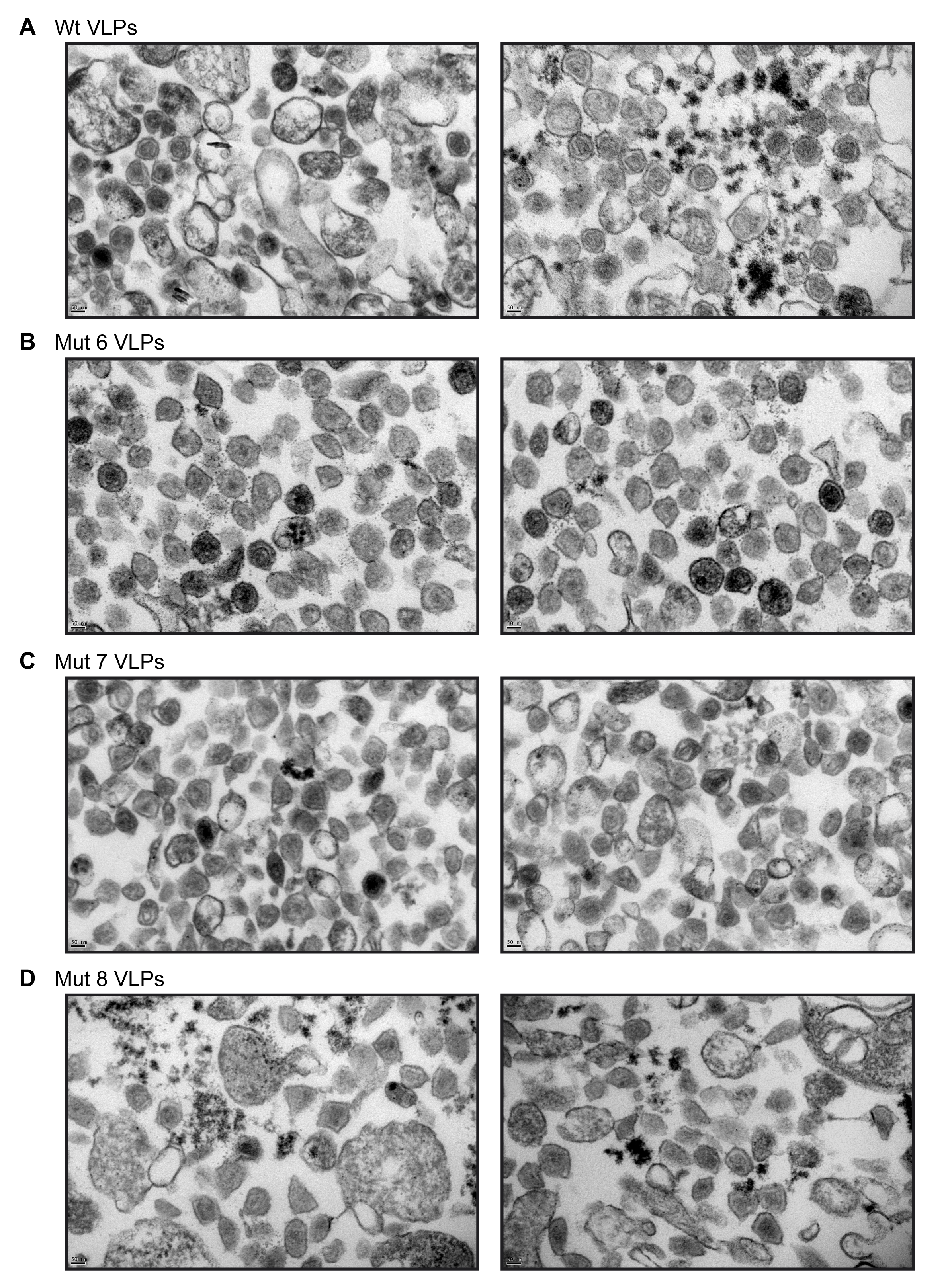

Supplement: Figure S3 — Additional electron micrographs of purified wild type and p12 mutant VLPs. Mo-MLV VLPs were purified and prepared for TEM, as in Fig. 4. Additional electron micrographs of (A) wild type, (B) p12 mutant 6, (C) p12 mutant 7 and (D) p12 mutant 8 VLPs are shown. All scale bars are 50 nm. (TIF) [file ppat.1004474.s003.tif]

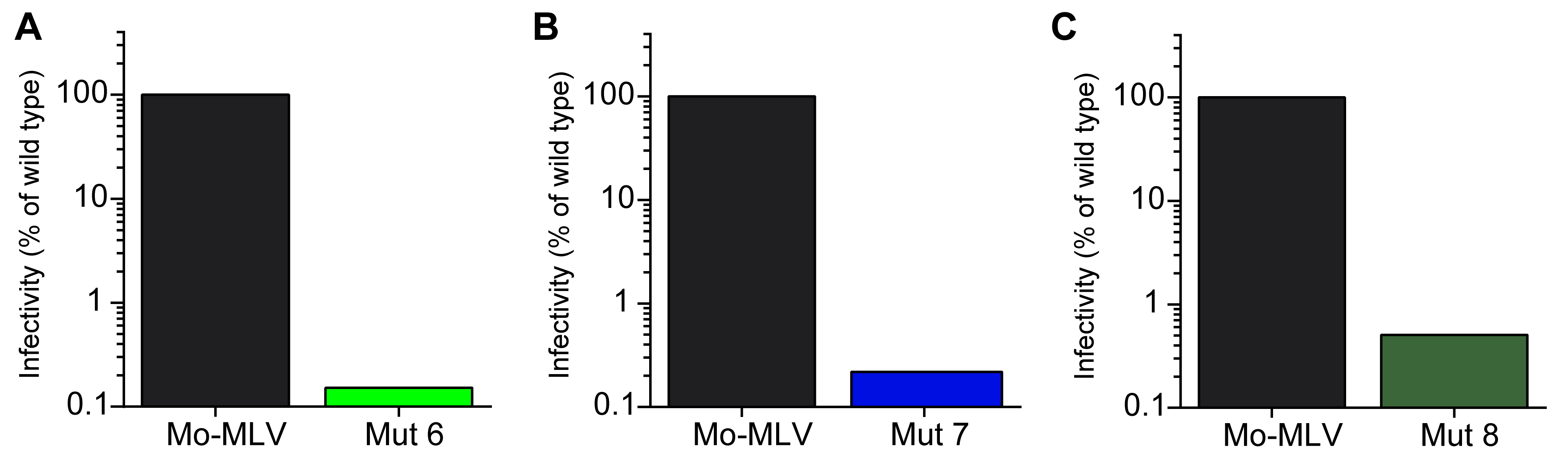

Supplement: Figure S4 — The infectivity of p12 mutant VLPs used for transmission electron microscopy. D17 cells were challenged with equivalent RT-units of the wild type and p12 mutant LacZ-encoding VLPs used in the TEM analysis (Fig. 4 and S3). Infectivity was measured by detection of beta-galactosidase activity in a chemiluminescent reporter assay and plotted as a percentage of wild type N-MLV infectivity. (A) Wild type and p12 mutant 6 VLPs, (B) Wild type and p12 mutant 7 VLPs and (C) Wild type and p12 mutant 8 VLPs. (TIF) [file ppat.1004474.s004.tif]

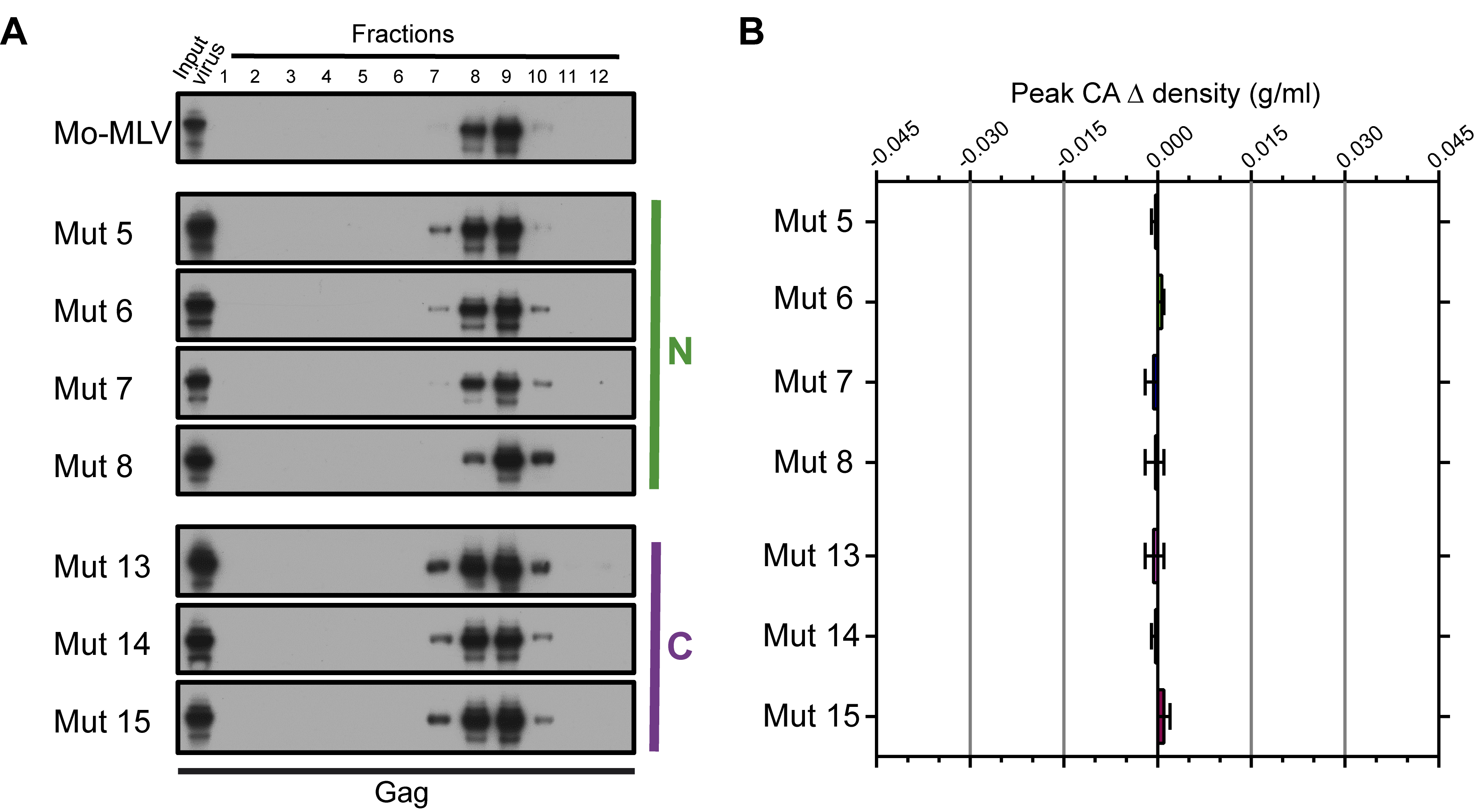

Supplement: Figure S5 — Migration profile of intact immature p12 mutant VLPs in an equilibrium gradient A mutation was introduced into PR to inactivate its activity (D32L, called PR-). (A) PR- wild type and p12 mutant VLPs were subjected to equilibrium sedimentation through a 10–42% (w/w) sucrose gradient (without detergent). Fractions were collected and analysed by immunoblotting using an anti-CA antibody. Representative immunoblots are shown (Fraction 1 is the top of the gradient). (B) For each experiment, the sucrose density of the fraction containing the peak CA signal was measured, and the change in density compared to peak CA fraction for wild type virions was calculated. The mean and range of three independent experiments are displayed in the histogram. (TIF) [file ppat.1004474.s005.tif]

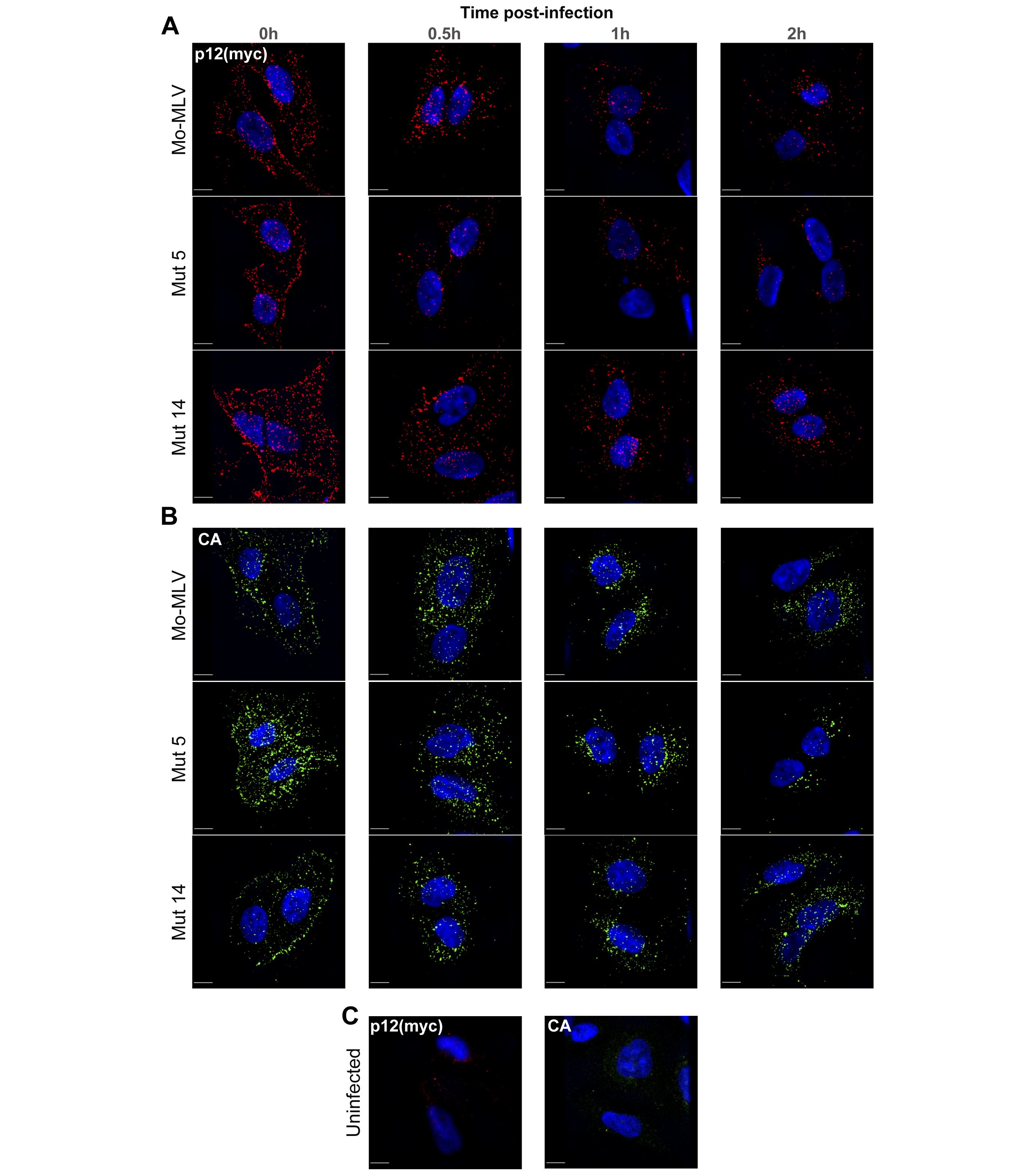

Supplement: Figure S6 — Immunofluorescence of p12 and CA in cells infected with Mo-MLV p12 mutant 5 and 14. U/R cells were challenged with ecotropic wild type, p12 mutant 5 or p12 mutant 14 Mo-MLV VLPs, containing a myc-tag in p12, by cold spinoculation (MOI 3). Cells were fixed at various times post-infection and stained with either an (A) anti-myc or (B) anti-CA antibody followed by a Cy3 (A) or FITC (B) -conjugated secondary antibody. The nuclear DNA was counterstained using DAPI (blue). (C) Images of uninfected U/R control cells fixed and stained as in (A) and (B). Images from the time course were captured using a spinning disk confocal microscope and representative images of cells from each time point are shown. All images are three dimensional acquisitions projected on a two dimensional plane. Images were processed using SlideBook. Scale bars are 10 µm. (TIF) [file ppat.1004474.s006.tif]

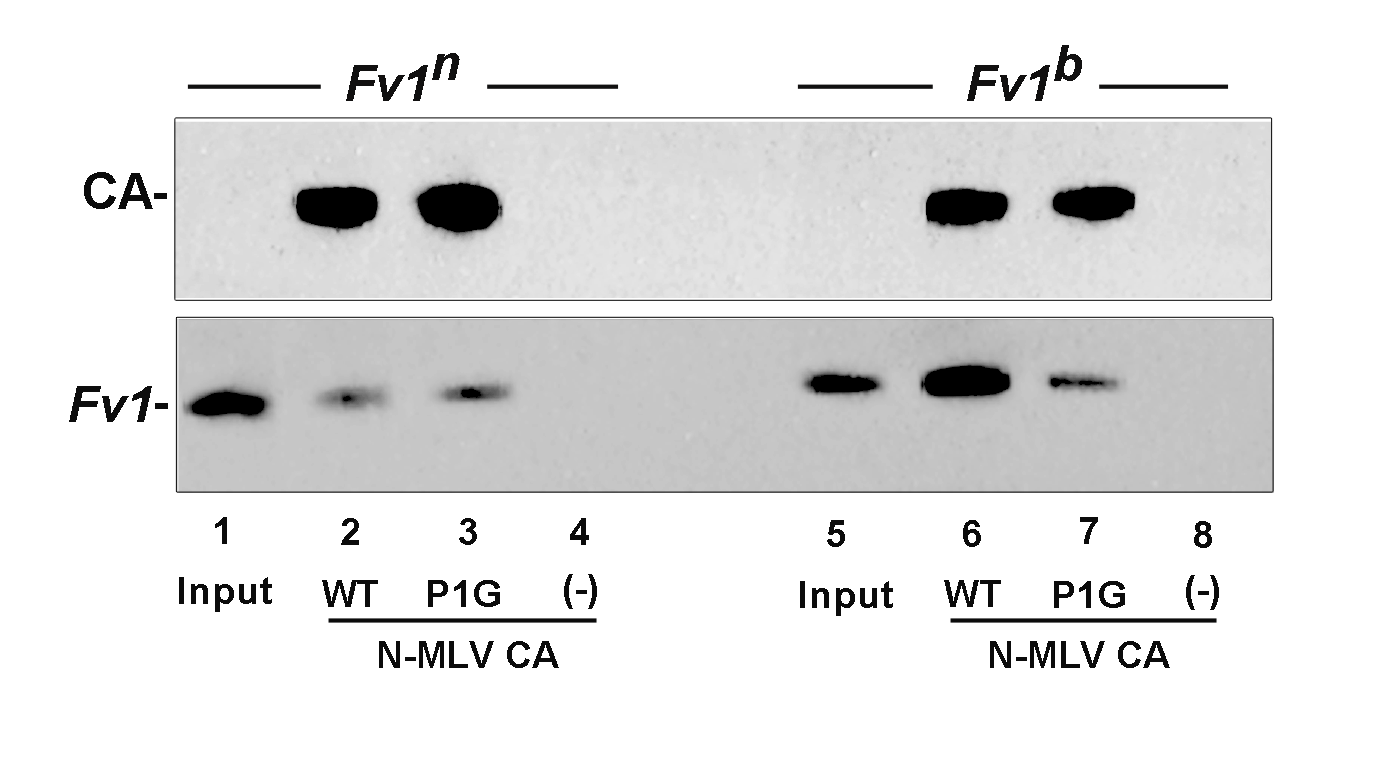

Supplement: Figure S7 — Binding of Fv1 to N-MLV CA-coated lipid nanotubes. Multimeric arrays of wild type (WT) and P1G mutant N-MLV CA were generated by immobilising the His-tagged purified proteins on lipid nanotubes comprising the Ni2+-chelating lipid, DGS-NTA. Cell lysates containing Fv1n (lanes 2–4) or Fv1b (lanes 6–8) were incubated with lipid nanotubes coated with WT CA (lanes 2 and 6), P1G CA (lanes 3 and 7) or no tubes (lanes 4 and 8) prior to centrifugation through a sucrose cushion. The pelleted material was resuspended in SDS-PAGE sample buffer and probed for CA and Fv1 by immunoblotting, using appropriate antibodies. Input, lanes 1 and 5, represents 1/16th dilution of cell extracts before incubation with CA-coated lipid nanotubes. (TIFF) [file ppat.1004474.s007.tiff]
