# Supplementary material for: The N-Terminus of Murine Leukaemia Virus p12 Protein Is Required for Mature Core Stability
Source: PLoS Pathog. 2014 Oct 30;10(10):e1004474. doi: 10.1371/journal.ppat.1004474 (PMC4214797; doi:10.1371/journal.ppat.1004474)
Supplement: Table S1 — Numbers of CA and p12 puncta counted in immunofluorescent studies. The mean number of CA or p12 puncta present per cell at time zero or 2 hours post-infection is shown +/− standard errors. The numbers of puncta at 2 hours post-infection were normalised to the numbers of puncta at time zero and these data are represented graphically in the bar chart in Figure 7F. (DOCX) [file ppat.1004474.s008.docx]

**Table S1:** **Numbers of CA and p12 puncta counted in immunofluorescent studies.**

| Time post infection (h) |  | Mo-MLV | Mut 5 | Mut 6 | Mut 7 | Mut 8 | Mut 14 |
| --- | --- | --- | --- | --- | --- | --- | --- |
| 0 | CA | 265 ±30 | 336 ±45 | 441 ±114 | 438 ±50 | 287 ±50 | 209 ±25 |
|  | p12 | 214 ±38 | 236 ±32 | 280 ±38 | 232 ±23 | 189 ±44 | 215 ±34 |
| 2 | CA | 255 ±26 | 79 ±11 | 97 ±17 | 99 ±14 | 99 ±14 | 152 ±15 |
|  | p12 | 122 ±13 | 57 ±13 | 26 ±6 | 45 ±13 | 24 ±4 | 127 ±17 |
